# Why Outcomes Diverge in Chronic Hepatitis B with Steatotic Liver Disease: Lessons from Within-Study Contrasts

**DOI:** 10.3390/medicina62101992

**Published:** 2026-10-15

**Authors:** Xiaoya Yang, Ziteng Wang, Hongshan Wei

**Affiliations:** 1 Department of Gastroenterology, Beijing Ditan Hospital, Capital Medical University, No. 8 Jingshun East Street, Chaoyang District, Beijing 100015, China; yangxiaoya777@163.com (X.Y.); wangzi_t@163.com (Z.W.); 2 HBV Infection, Clinical Cure and Immunology Laboratory for Clinical Medicine, Capital Medical University, No. 8 Jingshun East Street, Chaoyang District, Beijing 100015, China

**Keywords:** chronic hepatitis B, steatotic liver disease, metabolic dysfunction-associated steatotic liver disease, hepatic steatosis, cardiometabolic risk, hepatocellular carcinoma

## Abstract

*Background and Objectives*: The “friend or foe” debate in chronic hepatitis B (CHB) with steatotic liver disease (SLD) partly arises from treating hepatic fat, metabolic burden, and steatohepatitis as one exposure and interpreting different outcomes together. *Materials and Methods*: This narrative review applies an exposure–stage–context framework to literature identified in PubMed and Web of Science through 21 September 2026. We synthesized outcome evidence from 119 reports, including 14 detailed examples, using within-study comparisons of exposures, outcomes, and patient subgroups. *Results*: Within individual cohorts, associations with the same outcome could diverge: steatosis or an SLD diagnosis was associated with lower hepatocellular carcinoma (HCC) or mortality risk, whereas greater metabolic burden was associated with higher risk. Favorable antigen-related associations also coexisted with adverse fibrosis trajectories. Steatohepatitis was associated with more severe fibrosis and liver-related events, while metabolic abnormalities and prolonged glycemic exposure were associated with adverse hepatic outcomes. Studies of metabolic dysfunction-associated steatotic liver disease (MASLD) reported greater cardiovascular risk, and a study of hepatitis B virus-positive patients with nonalcoholic fatty liver disease (NAFLD) reported more prevalent kidney disease. Baseline fibrosis, patient selection, and antiviral treatment shape these associations; lower relative risks can coexist with a high absolute event burden. *Conclusions*: Current hepatic fat, cumulative metabolic exposure, and established injury therefore carry different prognostic meanings. Their separate assessment helps explain part of the apparent paradox, while adverse steatosis findings and discrepancies in similar settings remain unresolved. The evidence is predominantly observational.

## 1. Introduction

Chronic hepatitis B (CHB) remains a major cause of cirrhosis, hepatocellular carcinoma (HCC), and liver-related mortality [1,2]. Sustained viral suppression has expanded long-term care to encompass fibrosis, HCC risk, and metabolic comorbidities [3,4,5,6]. Concurrently, the global burden of steatotic liver disease (SLD) has increased alongside obesity and type 2 diabetes [7,8,9,10,11,12]. Hepatic steatosis is common in CHB, with prevalence estimates varying by diagnostic method, case definition, and population [13,14,15]. Understanding this coexistence is therefore relevant to both hepatic and systemic risk assessment.

Experimental and translational studies implicate hepatic fat, metabolic inflammation, and interferon pathways in hepatitis B virus (HBV) replication [16,17,18,19]. These mechanisms offer plausible explanations for antigen-related findings, but fibrosis, cancer, and systemic disease also reflect accumulated injury and metabolic exposure. The clinical question is therefore how these processes contribute to different outcomes over the course of CHB.

SLD is the umbrella term, and its diagnostic categories identify different patient groups [20,21,22,23]. Nonalcoholic fatty liver disease (NAFLD) historically required steatosis with exclusion of significant alcohol use and other causes of liver disease, although CHB studies used varying criteria. Metabolic dysfunction-associated fatty liver disease (MAFLD) requires steatosis plus overweight/obesity, type 2 diabetes, or at least two specified metabolic abnormalities and permits coexisting liver diseases [20,24,25]. Metabolic dysfunction-associated steatotic liver disease (MASLD) requires steatosis, at least one cardiometabolic risk factor, and alcohol intake within the MASLD range; CHB may coexist [22,23]. Metabolic dysfunction and alcohol-associated liver disease (MetALD) combines metabolic risk with higher alcohol intake within specified ranges [22,26]. Historical NAFLD findings inform fat-related and metabolic risk. Their applicability to MAFLD depends on the corresponding metabolic criteria; applicability to MASLD also requires compatible alcohol exposure. We retain each study’s original exposure label and HBV population definition.

Some cohorts associate SLD with favorable antigen-related findings [27,28,29], while others associate steatohepatitis or metabolic dysfunction with adverse hepatic outcomes [30,31,32,33]. HCC findings are particularly heterogeneous [34,35,36,37,38,39]. The “friend or foe” formulation captures this tension [40], but a single verdict on “fatty liver” conflates fat quantity, inflammatory injury, and metabolic disease. It also obscures the possibility that the same exposure has different associations with antigen clearance, fibrosis, and systemic outcomes.

**Figure 1:**
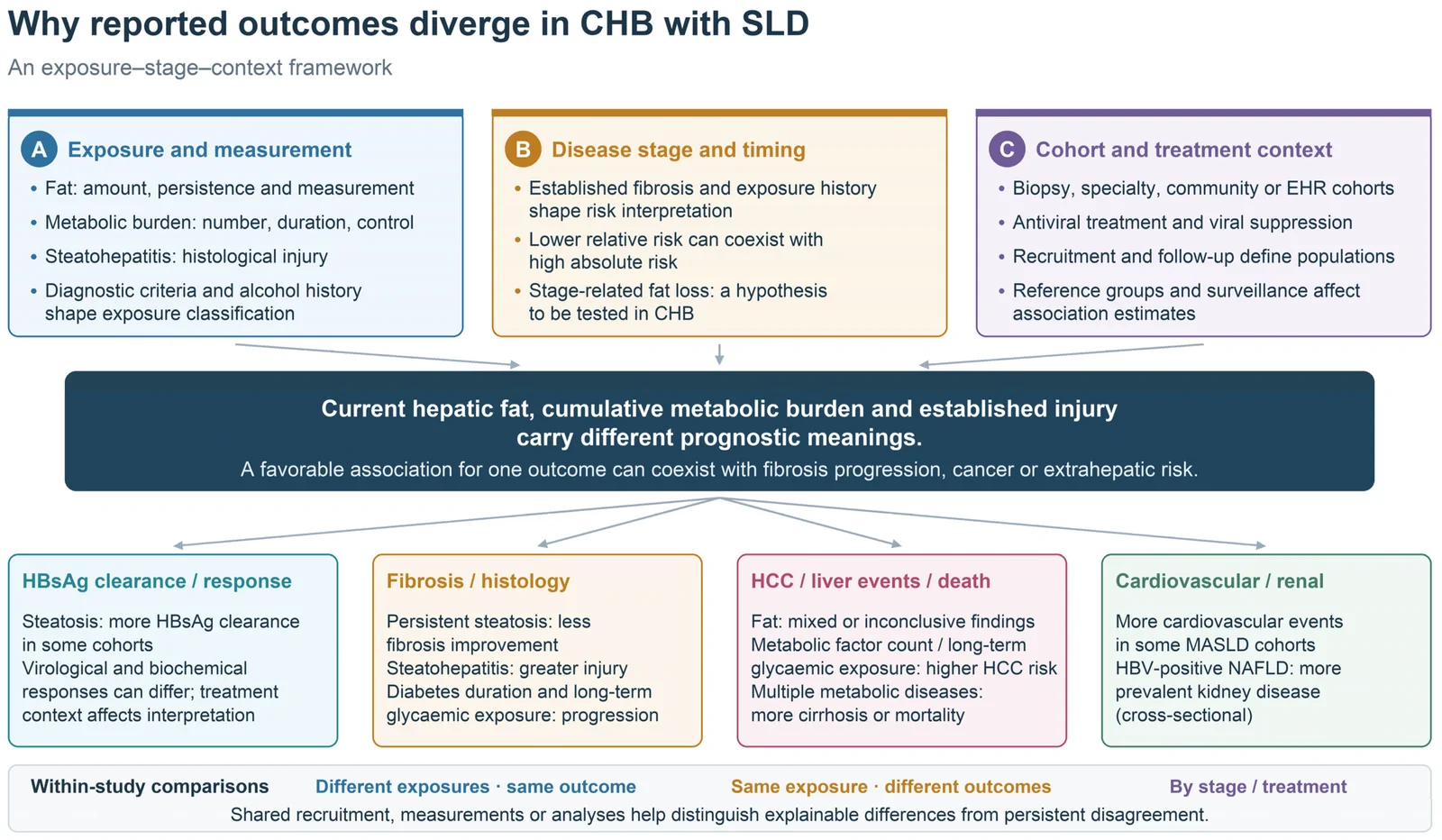
An exposure–stage–context framework for interpreting divergent outcomes in CHB with SLD. Abbreviations: CHB, chronic hepatitis B; HBV, hepatitis B virus; HBsAg, hepatitis B surface antigen; HCC, hepatocellular carcinoma; SLD, steatotic liver disease; MASLD, metabolic dysfunction-associated steatotic liver disease; NAFLD, nonalcoholic fatty liver disease; EHR, electronic health record. Arrows indicate interpretive relationships; the findings are primarily observational.

Previous “friend or foe” reviews distinguish steatosis from metabolic dysfunction and discuss disease severity, treatment, and reverse causation [40,41,42,43,44]. This structured narrative review examines how exposure definitions, disease stage, patient selection, treatment context, and outcome assessment help explain divergent prognostic findings on SLD and metabolic dysfunction in CHB. We examine within-study comparisons that change the interpretation: hepatic fat and metabolic burden assessed against the same outcome, one exposure related to different outcomes, or associations compared by disease stage and treatment. Shared recruitment, measurements, or analytical methods make these contrasts more informative about the source of disagreement than juxtaposing estimates from unrelated cohorts. The exposure–stage–context framework (Figure 1) distinguishes apparent contradictions that these differences can explain from discrepancies that remain unresolved. Cross-cohort contrasts in evidence syntheses are examined separately. This analysis emphasizes the distinct prognostic meanings of current hepatic fat, accumulated metabolic exposure, and established injury, and identifies prospective tests of this interpretation.

## 2. Methods

### 2.1. Literature Search and Handling of Duplicate and Overlapping Data

This structured narrative review used purposive evidence selection and qualitative synthesis rather than exhaustive study inclusion or quantitative pooling. PubMed and Web of Science Core Collection were searched through 21 September 2026, without language or publication-type restrictions. PubMed covered all available years; coverage in the selected Web of Science indexes began in 1992 for the Science Citation Index Expanded, 1985 for Current Chemical Reactions, and 1993 for Index Chemicus.

Hepatitis B, HBV, and CHB terms were combined using AND with terms for hepatic steatosis, NAFLD, MAFLD, MASLD, steatohepatitis, and cardiometabolic factors. A representative combination was (hepatitis B OR HBV OR CHB) AND (steatosis OR NAFLD OR MAFLD OR MASLD OR diabetes OR obesity). PubMed used Medical Subject Headings and title/abstract fields; Web of Science used Topic fields and truncation such as diabet* and obes*. Supplementary Methods S1 provides the complete search strategies.

We also checked the manuscript reference list, the bibliography assembled during revision, and all 46 references in the review by Huang and Tseng (2026) [40]. Duplicate records were consolidated using PubMed identifiers, digital object identifiers, and publication details. Population overlap was assessed from study centers, recruitment periods, participants, and follow-up. Different analyses from shared cohorts could be retained, with their relationships recorded to avoid treating them as independent replication. When a conference abstract and journal article reported the same analysis, we used the journal results. Conference records with unconfirmed links were not used as independent outcome evidence.

### 2.2. Screening and Use of Reports

PubMed yielded 7025 records and Web of Science 7399, totaling 14,424. Consolidation removed 5116 duplicate records, leaving 9308; 39 additional records brought the total assessed to 9347.

Candidates were identified from titles, available abstracts, and existing assessments. Unclear population, exposure, or comparison information prompted checks of the relevant methods, results, tables, or Supplementary Material. After assessment, 3926 records were excluded as outside scope, and two were retracted reports or associated notices; 4938 were classified as potential background, 223 remained of uncertain eligibility, and 258 reports met the comparison criteria.

Of the 258 qualifying reports, 119 contributed to outcome synthesis, 38 provided background, 11 lacked key information needed for interpretation, and 90 were not selected, for the reasons listed in Table S1E. Among the 119 outcome reports, 14 are presented in detail in Table 1, and the other 105 contribute through the text or thematic summaries.

Figure S1 shows the counts and dispositions; Table S1 records the decisions and use of each report. Potential background included relevant single-association, diagnostic, and mechanistic studies, as well as guidelines, terminology statements, and narrative reviews.

### 2.3. Eligibility Criteria for Comparisons

Original human studies had to provide extractable CHB or source-defined HBV-positive data on associations of hepatic steatosis, a study-defined SLD category, or steatohepatitis with relevant outcomes. Studies of cardiometabolic factors within CHB–SLD populations were also in scope. Reports required at least one comparison: (1) different steatotic or cardiometabolic exposures, diagnostic definitions or exposure changes evaluated against a common outcome; (2) a relevant exposure evaluated across different outcome domains, including virological, serological, biochemical, histological, hepatic, extrahepatic or mortality outcomes; or (3) associations compared across disease stages, treatment, viral status, diagnostic methods or recruitment settings. Evidence syntheses had to provide extractable CHB–SLD comparisons. Eligibility did not depend on result direction, statistical significance, or sample size. Baseline descriptions, covariate adjustment, and matching were treated as analytical features.

### 2.4. Outcome Synthesis and Selection of Illustrative Reports

Findings were synthesized by exposure, outcome, disease stage, and clinical setting, including conflicting and inconclusive results. We assessed study limitations qualitatively, considering selection bias, residual confounding, exposure and outcome misclassification, measurement timing, treatment context, follow-up duration, and potential cohort overlap. Tables S1A and S1B record the use of each report, Table S1D summarizes additional comparative studies [52,53,54,55,56,57,58,59,60,61,62,63,64,65,66,67,68,69,70,71,72,73,74,75,76,77,78,79,80,81,82,83,84,85,86,87,88,89,90,91,92,93,94,95,96,97,98,99,100,101,102,103,104,105,106,107,108,109], and Table S2 presents examples of supporting background studies [110,111,112,113,114].

The 14 reports in Table 1 were selected as illustrative examples because their comparisons addressed the review questions; exposure and reference groups, outcomes and analyses were clearly described; and the reports complemented one another across exposure definitions, metabolic burden, exposure changes, outcome domains, disease stage and treatment. Detailed presentation alone does not confer additional evidentiary weight. Table S1C gives the reason for each choice.

The authors used ChatGPT-5.5 (OpenAI; https://chatgpt.com/, accessed on 30 June 2026) for English language editing and grammatical revision. The authors reviewed and edited the output and take responsibility for the final content.

## 3. Comparisons Within Studies and Evidence Syntheses

### 3.1. Evidence and Illustrative Comparisons

Outcome findings were drawn from 119 reports, of which 14 are presented in detail in Table 1 and 105 contribute through the text or thematic summaries.

### 3.2. What the Comparisons Show

Current hepatic fat and metabolic burden can point in opposite prognostic directions within the same cohort. Huang et al. [35] associated steatosis with lower HCC risk and each additional metabolic risk factor with higher risk. For mortality, Huang et al. [32] similarly found an inverse SLD association in the matched population but higher risk with MASLD within the SLD group. A binary SLD label therefore obscures clinically relevant differences in metabolic severity. Lin et al. [36] associated MASLD with HCC but found an inconclusive risk-factor-count comparison within MASLD; Peleg et al. [45] associated steatosis itself with death or any cancer, and Li et al. [46] associated both diabetes and steatosis severity with fibrosis. Hepatic fat and metabolic burden warrant separate assessment: their associations may oppose one another, or both indicate adverse outcomes.

A favorable antigen-related association can coexist with structural injury. In Mak et al. [28], baseline steatosis defined by controlled attenuation parameter (CAP) was associated with hepatitis B surface antigen (HBsAg) clearance, while persistent severe steatosis accompanied more progression in liver-stiffness stage in an unadjusted comparison. Yang et al. [51] associated persistent steatosis during treatment with less improvement on paired biopsies. Chau et al. [48] found different response patterns for biochemical and virological endpoints. Antigen clearance and viral suppression describe one aspect of prognosis; overall assessment also requires structural, cardiovascular and renal outcomes [49,50].

Relative associations and absolute incidence answer different questions. Wong et al. [39] found lower adjusted hazards of HCC, cirrhosis and death with fatty liver in a weighted individual-patient-data analysis, yet biopsy-selected fatty-liver cohorts had high absolute HCC incidence. Lower relative risk can therefore coexist with a substantial absolute event burden. The higher pooled HCC odds reported by Mao et al. [37] also show that interpretation depends on the populations, effect measures, and adjustment used in a synthesis. These analyses include overlapping cohorts, and their diagnostic-method comparisons partly involve different populations.

### 3.3. Testable Propositions

First, cumulative metabolic exposure may add prognostic information beyond current hepatic fat, fibrosis stage, and established CHB predictors. The relevant exposure includes the number, duration, and control of metabolic abnormalities over time.

Second, inverse fat–HCC associations may partly reflect disease stage and prior exposure. If detectable fat declines during progression, the group without current steatosis may become enriched for patients with previous steatosis and established injury. This temporal explanation remains unconfirmed in CHB.

Third, under sustained viral suppression, fibrosis stage and metabolic trajectories may contribute more to residual risk prediction than current hepatic fat. Their relative prognostic contributions may therefore differ before and after viral control.

## 4. Steatotic and Metabolic Exposures

The prognostic meaning of “fatty liver” depends on what is measured. Fat quantity, metabolic disease and steatohepatitis capture different aspects of CHB–SLD [20,22,23]; alcohol history, diagnostic coding and measurement timing further determine who enters each category [29,115]. Table 2 sets out these distinctions.

### 4.1. Hepatic Fat Deposition

Ultrasonography, CAP, magnetic resonance imaging–proton density fat fraction (MRI-PDFF), and histology assess different aspects of hepatic fat [7,10,116,117,118,119]. Ultrasonography provides qualitative or semiquantitative assessment, CAP estimates steatosis, and MRI-PDFF quantifies liver fat fraction [117,119]. Histology additionally characterizes inflammation, ballooning, and fibrosis [116,118]. Thus, imaging-defined steatosis primarily represents fat deposition, whereas histological steatohepatitis represents fat-associated injury.

### 4.2. MAFLD/MASLD and Metabolic Abnormalities over Time

MAFLD and MASLD combine steatosis with metabolic criteria through different diagnostic algorithms [20,21,22,23]. A shared diagnosis can nevertheless encompass markedly different numbers, severity, and duration of metabolic abnormalities [7,22]. These differences matter when comparing prognosis within a MASLD population or between studies using the same label.

Risk-factor counts describe baseline burden; incident disease and repeated glycemic measurements capture subsequent exposure [32,33,122]. Body mass index alone misses part of this variation, including metabolic risk in non-obese patients with study-defined NAFLD [127].

### 4.3. Alcohol Exposure and MetALD

Alcohol thresholds distinguish NAFLD, MASLD and MetALD, whereas MAFLD permits coexisting alcohol-related liver disease [20,21,22,26]. Lam et al. [121] reported associations of measured alcohol exposure and metabolic syndrome with fibrosis in CHB, but did not classify patients using contemporary MetALD criteria. Direct prognostic evidence for CHB with MetALD remains limited; recommendations to record alcohol amount, pattern, and timing follow general classification guidance [26].

### 4.4. Histological Steatohepatitis

Histological steatohepatitis comprises steatosis, lobular inflammation, and hepatocellular ballooning; fibrosis is staged separately [116,118]. We use nonalcoholic steatohepatitis (NASH) for historical diagnoses and metabolic dysfunction-associated steatohepatitis (MASH) for studies applying the contemporary criteria [22]. In CHB, interpretation requires consideration of both viral and metabolic injury, fibrosis stage, and antiviral treatment [128].

### 4.5. SLD Identified from Electronic Health Records

SLD identified from electronic health record (EHR) codes reflects both the disease and how it is recorded in clinical practice. Imaging, metabolic assessment, testing frequency, and documentation all influence which patients receive a diagnosis [29]. Agreement with CAP-defined steatosis, MRI-PDFF, MASLD, or histological NASH/MASH depends on the coding algorithm and its validation. Interpretation therefore requires the code lists and methods used to identify outcomes.

### 4.6. Changes in Liver Fat in Advanced Disease

Fat loss with fibrosis progression remains a hypothesis in CHB. Supporting observations come from NASH/cryptogenic cirrhosis [115,125] and cross-sectional CHB comparisons showing lower MRI-PDFF with cirrhosis [126]. Serial measurements are needed to demonstrate within-person fat loss. Such loss could place patients with previous steatosis and advanced injury in the group without current fat, contributing to inverse associations through reverse causation.

## 5. Cohort Selection and Treatment Context

Diagnostic selection changes the baseline risk carried by a “fatty liver” group. Biopsy cohorts often recruit patients with abnormal aminotransferases, diagnostic uncertainty, or suspected advanced disease; community and EHR cohorts capture a broader clinical spectrum. Wong et al. [39] reported substantially higher 10-year HCC incidence in biopsy-based fatty-liver cohorts than in cohorts using non-invasive diagnosis. Within biopsy-based cohorts, patients with fatty liver were more likely to have cirrhosis at baseline than those without fatty liver. Patient selection and established injury help explain why inverse associations in a broader synthesis can coexist with high event rates in biopsy populations.

Antiviral treatment changes the viral contribution to prognosis, but treated cohorts still differ in baseline fibrosis, duration of suppression, and metabolic disease. Mao et al. [37] reported an imprecise HCC association in nucleos(t)ide analogue-treated studies (pooled odds ratio (OR) 1.05, 95% confidence interval (CI) 0.62–1.77), while individual treated cohorts reported varying fat-related and metabolic associations [34,38,48,129]. Comparisons between treated cohorts therefore need to distinguish baseline injury, duration of viral suppression, and metabolic burden.

## 6. Virologic and Serologic Outcomes

The interpretation of antigen-related findings depends on how clearance is established. Confirmed HBsAg seroclearance describes a longitudinal event; EHR records of antigen status also reflect testing and documentation. Virological suppression and biochemical response describe different aspects of treatment outcome.

Mak et al. [28] observed HBsAg seroclearance in 22 of 330 patients with virologically quiescent CHB; baseline CAP-defined steatosis was associated with clearance (Cox hazard ratio (HR) 3.246, 95% CI 1.278–8.243). Huang et al. [27] reported associations of MASLD with seroclearance and seroconversion in untreated hepatitis B e antigen (HBeAg)-negative CHB. Yendewa et al. [29] found lower recorded proportions of detectable HBsAg and HBeAg with EHR-defined SLD.

Chang et al. [130] studied 720 Korean patients with quiescent CHB. Associations of baseline steatosis with HBsAg loss, stiffness-stage progression, and HCC were inconclusive; the univariate HR for HBsAg loss was 1.440 (95% CI 0.908–2.285). HBsAg loss required one negative result, whereas Mak required repeated confirmation. The studies also differed in the timing of exposure assessment.

Treatment cohorts distinguish biochemical from virological response. In the matched Real-World Evidence from the Global Alliance for the Study of HBV (REAL-B) analysis, Chau et al. [48] reported adjusted subdistribution hazard ratios of 0.77 (95% CI 0.68–0.88) for biochemical response and 0.97 (0.86–1.11) for virological response with SLD. Li et al. [131] assessed antigen and biochemical outcomes during pegylated-interferon add-on therapy; Chen et al. [132] reported inconclusive differences in 48-week virological response across steatosis categories. In Li et al.’s Stanford cohort [133], the adjusted HRs comparing NAFLD with its absence were 1.13 (95% CI 0.86–1.49) for viral suppression and 1.02 (0.67–1.55) for biochemical response. Treatment regimens differed across reports, and the Stanford and REAL-B cohorts may include some of the same patients.

In a related REAL-B analysis, metabolic disease with or without steatosis was associated with lower biochemical response; the estimate for steatosis alone was inconclusive [134]. In a biopsy-selected cohort with normal alanine aminotransferase (ALT), MASLD was associated with higher one-year HBV DNA clearance and more histological injury at baseline [135]. Better virological response can coexist with greater pre-existing tissue injury; longer-term benefit should be assessed through viral control, fibrosis and metabolic disease together. Supplementary Table S1D summarizes other nucleos(t)ide analogue and pegylated-interferon findings by endpoint and follow-up.

## 7. Fibrosis and Liver Disease Progression

Fibrosis studies examine baseline injury and recovery during treatment. Baseline biopsies compare established injury in patients with and without steatosis, whereas paired biopsies and serial measurements assess persistent steatosis in relation to subsequent recovery. Liver stiffness also reflects inflammation and requires separate interpretation from histological change.

Cross-sectional biopsy findings vary. Yao et al. [120] reported an association of NAFLD with less severe fibrosis in treatment-naïve CHB, whereas Huang et al. [136] found an association of NASH with significant fibrosis. Li et al. [46] associated both diabetes and greater steatosis severity with significant fibrosis; another report by Li et al. [137] described greater inflammation and fibrosis with NAFLD despite normal ALT. In the smaller cohort of Yilmaz et al. [138], differences in histological activity and fibrosis across steatosis groups were inconclusive. In the North American Hepatitis B Research Network, steatohepatitis was associated with a higher prevalence of Ishak stage ≥3 than steatosis alone (adjusted relative risk 2.89, 95% CI 1.54–5.41), after adjustment for age, sex, ALT, and HBV DNA [123]. In a Thai biopsy cohort [139], steatohepatitis was associated with advanced fibrosis (adjusted odds ratio (aOR) 3.45, 95% CI 1.11–10.7; reference: absence of steatohepatitis). In its treated subset, combined viral and biochemical response at week 48 occurred in 6/14 patients with steatohepatitis and 52/98 without it (unadjusted *p* = 0.475).

Longitudinal studies assess changes in steatosis alongside changes in fibrosis. In an unadjusted comparison, Mak et al. [28] reported liver-stiffness-defined stage progression in 41.3% of patients with persistent severe steatosis and 23.0% with persistent absence of steatosis over three years (*p* = 0.05). Yang et al. [51] associated persistent steatosis during antiviral therapy with lower odds of histological improvement: aOR 0.244 (95% CI 0.090–0.665) for inflammation and 0.180 (0.064–0.507) for fibrosis, relative to persistent absence of steatosis.

Two other paired-biopsy studies examined changes in steatosis during treatment [140,141]. In the 104-week EFFORT subgroup, persistent and newly developed steatosis were associated with lower odds of fibrosis improvement than persistent absence of steatosis (aOR 0.247, 95% CI 0.077–0.800, and 0.185, 0.058–0.588, respectively), adjusting for baseline Ishak stage [140]. During 78 weeks of entecavir-based therapy, the corresponding estimates were 0.380 (0.145–0.996) for persistent steatosis and 0.936 (0.340–2.575) for newly developed steatosis [141]. Across these three treated biopsy reports, persistent steatosis was associated with less fibrosis improvement, whereas findings for newly developed steatosis differed. Treatment, adjustment, and regression definitions varied, and fat and fibrosis changes were assessed over the same interval.

Wong et al. [124] associated incident metabolic syndrome with a ≥30% increase in liver stiffness. Hong et al. [31] reported greater prevalence of biopsy-defined fibrosis with more cardiometabolic risk factors; Lv et al. [142] assessed diabetes and hypertension separately in patients with HBV-positive MAFLD. In the HBV subgroup studied by Huang et al. [143], differences in inflammation and fibrosis were inconclusive across the overlapping MAFLD, NAFLD and steatosis-without-metabolic-risk groups, as defined in that study. In Gezer et al. [144], Ishak stage ≥ 3 occurred in 11.6%, 17.1% and 17.5% of MASLD patients with one, two and at least three factors, respectively (unadjusted *p* = 0.349). The same MASLD diagnosis encompasses different exposure histories, some of which a risk-factor count captures. The prognostic contributions of individual disorders, their duration, and their accumulation require separate assessment.

Findings also depend on how fibrosis is measured [47,145]. In a CHB study with biopsy assessment, MRI-PDFF remained associated with liver stiffness within histological stages F1 and F2; the area under the receiver operating characteristic curve for identifying stage ≥ 2 was 0.789 in both MRI-PDFF groups (<5% versus ≥5%; *p* = 0.990) [146]. Con et al. [47] associated steatosis with an estimated 9.4% decrease in liver stiffness at eight years (95% CI 1.3–16.8% decrease); the corresponding estimate for the metabolic composite was +0.8% (−6.1% to +8.4%). Other reports examine fibrosis measures and diagnostic performance in specific patient groups [46,147,148,149].

Patmore et al. [145] associated MASLD with less frequent decreases and more frequent increases in stiffness stage, including after antiviral initiation. In another five-year cohort, differences in aspartate aminotransferase-to-platelet ratio index trajectories were accompanied by imprecise clinical-event estimates, with only eight cirrhosis events [150]. Baseline fibrosis and subsequent recovery require separate assessment: less injury at entry can coexist with limited improvement during follow-up. The paired-biopsy findings support persistent steatosis as a marker of incomplete structural recovery. Establishing its contribution requires separating fat changes from concurrent changes in fibrosis and metabolic disease. Table S1D provides the remaining biopsy and serial-marker findings.

Choi et al. [30] examined clinical events in groups defined jointly by NASH and advanced fibrosis. Relative to the NASH−/advanced-fibrosis− group, the adjusted hazard ratio (aHR) was 4.81 (95% CI 2.59–8.95) in patients with both conditions and 2.30 (1.31–4.05) in those with advanced fibrosis alone. The MAFLD report [24] analyzed 1076 participants from the same 1089-person cohort after excluding 13 with insufficient metabolic information. Wu et al. [17] provide cross-sectional human tissue findings in CHB with MASLD, together with experimental evidence on viral mechanisms.

Brouwer et al. [151] assessed steatohepatitis, as defined in the original study, in a Rotterdam biopsy cohort. Age-adjusted associations were imprecise for death (HR 1.9, 95% CI 0.8–4.5) and HCC (1.6, 0.5–5.4); few events limited further adjustment. The study permitted alcohol exposure. Recruitment periods overlapped with the Rotterdam component of the Choi and van Kleef studies, so these reports may include some of the same patients.

In a separate treated biopsy cohort, steatohepatitis was associated with mortality/transplantation and liver-related events, whereas estimates for simple steatosis were imprecise. The steatohepatitis–mortality estimate was also imprecise in early fibrosis [152]. These findings place inflammatory injury and established fibrosis at the center of prognosis, beyond the presence of fat alone.

## 8. Hepatocellular Carcinoma Risk and Prognosis

HCC risk assessment requires examining whether the fat–HCC association persists after accounting for metabolic burden and fibrosis in the same population. Established CHB determinants—viral factors, cirrhosis, treatment and surveillance—remain central [3,4,5,6,153].

The opposing pooled HCC findings [37,39] illustrate the limits of assigning one effect to SLD across populations. Individual studies provide more specific comparisons of fat, metabolic burden, and disease stage.

Adalı et al. [34] reported higher HCC incidence with ≥3 cardiometabolic risk factors in a univariable analysis; the confidence intervals for MASLD and steatosis included 1. In treated CHB, Wang et al. [38] found an inverse SLD–HCC association alongside higher risk with ≥2 cardiometabolic factors. Patients without SLD who had ≥2 factors had the highest observed incidence. Lin et al. [36] associated MASLD with HCC in the overall cohort; among patients with MASLD, the comparison of ≥3 versus <3 factors yielded a log-rank *p* value of 0.066, with 36 HCC events overall. The populations and reference groups differed across these studies. In virologically suppressed CHB, Oh et al. reported lower HCC incidence with higher CAP among patients with advanced chronic liver disease, defined using a 10-kPa liver-stiffness cutoff; the corresponding comparison below that stiffness threshold was inconclusive [154].

Yun et al. [155] studied 197,346 Korean participants with CHB in a nationwide claims-based cohort. MAFLD, defined using a fatty liver index and metabolic criteria, was associated with HCC (aHR 1.36, 95% CI 1.31–1.41) and all-cause mortality (1.23, 1.18–1.29), each relative to non-MAFLD, after adjustment for age, sex, cirrhosis, antiviral therapy and lifestyle variables.

Yu et al. reported an association of MAFLD with greater HBsAg seroclearance and an HCC estimate close to 1. Diabetes, obesity and higher metabolic-abnormality counts were associated with greater HCC risk [156]. Toh et al. found a positive adjusted association between obesity and HCC, whereas the adjusted confidence intervals for diabetes, hypertension and steatosis included 1 [157]. During sustained HBV suppression, Cho et al. associated radiologic NAFLD with incomplete biochemical response and higher subsequent HCC risk [158]. Tan et al. [159] assessed cirrhosis and HCC in separate groups at risk; ten HCC events and wide confidence intervals limited precision. Peleg et al. [45] evaluated the broader composite of mortality or any cancer.

Wu et al. [160] associated code-defined SLD with lower HCC incidence in men and higher incidence in women, with sex–SLD interaction tests supporting this difference in antiviral-treated and untreated cohorts (both *p* < 0.0001). In the treated cohort, adjusted subdistribution HRs were 0.85 (95% CI 0.74–0.96) in men and 1.42 (1.17–1.73) in women, each relative to patients of the same sex without SLD. The models accounted for competing mortality. The opposing sex-specific estimates show how an overall SLD association can conceal heterogeneity within a shared study design.

Lam et al. [121] used structural equation models to examine relationships among metabolic syndrome, alcohol, fibrosis, and HCC. The estimated direct metabolic–HCC association differed between the full cohort and the advanced-fibrosis subset. These findings suggest that the relationship may vary with disease stage; causal mediation remains to be established. The high HCC incidence in patients without SLD but with multiple metabolic risk factors [38] also highlights the importance of the reference group. Absence of current fat can coexist with substantial metabolic burden and established injury. An inverse fat association can therefore accompany clinically important HCC risk; fat status, metabolic history, and fibrosis give complementary information.

Outcomes after HCC treatment address a different clinical question from HCC incidence. After resection of HBV-related HCC, Yoon et al. observed an unadjusted survival advantage with NAFLD that attenuated after adjustment (aHR 0.94, 95% CI 0.51–1.73); comparisons of recurrence-free and overall survival by NASH status were also inconclusive [161]. Among 1115 patients receiving immune checkpoint inhibitors for HBV-related HCC, survival comparisons between HBV alone and HBV with MASLD were inconclusive after weighting; within the HBV-alone group, low levels of high-density lipoprotein cholesterol were associated with poorer survival and overweight with better overall survival [162]. After liver transplantation for HBV-related HCC, pre-transplant NASH was associated with more HBV reactivation; the NASH–recurrence-free-survival comparison was adverse among patients with reactivation and inconclusive among those without it [163]. The attenuation after adjustment in the resection cohort suggests that baseline patient differences contributed to the apparent survival advantage. Once HCC is established, tumor burden, liver reserve, and treatment selection may change the prognostic meaning of metabolic characteristics.

## 9. Clinical Events, Mortality, and Extrahepatic Outcomes

Mortality and extrahepatic outcomes can differ in direction from hepatic outcomes. Within-cohort comparisons further distinguish the risks associated with an SLD diagnosis from those associated with metabolic disease and its duration [32,33,122].

Huang et al. [32] reported a lower mortality hazard for SLD versus its absence in a matched sample (HR 0.62, 95% CI 0.53–0.74). Within the SLD population, MASLD was associated with higher mortality than SLD without the cardiometabolic criteria required for MASLD (aHR 1.79, 1.24–2.58). Related National Taiwan University Hospital reports examined antigen outcomes, HCC, and cirrhosis in cohorts with overlapping recruitment settings [27,33,35].

Huang et al. [33] associated metabolic dysfunction with cirrhosis-related outcomes alongside inverse steatosis associations. van Kleef et al. [24] associated MAFLD with a composite of decompensation, HCC, transplantation, or death (aHR 2.00, 95% CI 1.26–3.19). Peleg et al. [45] instead associated steatosis itself with a mortality-or-cancer composite. In a cohort of 439 patients with HBV-related cirrhosis, including 332 with decompensation, MAFLD was associated with greater mortality and further decompensation before and after propensity-score matching [164].

Metabolic history also distinguishes patients who share the same diagnosis. In CHB with diabetes, Mak et al. [122] associated diabetes duration and repeated glycemic exposure with HCC and fibrosis progression. In treated CHB, Huang R et al. [129] associated ≥2 metabolic diseases with cirrhosis, all-cause mortality and non-liver mortality; diabetes was also associated with HCC. The REAL-B treatment-response and clinical-event reports drew from overlapping recruitment settings [48,129]. Duration, control, and incident metabolic disease [33,122] add exposure history beyond a baseline diagnosis, suggesting that cumulative burden may provide additional prognostic information. Whether trajectories of weight, blood pressure, and lipids contribute similarly requires prospective study.

Yendewa et al. [29] associated EHR-defined SLD with greater observed fibrosis risk but lower risks of mortality, HCC, and end-stage liver disease. These associations may be influenced by broad diagnostic coding, testing and documentation patterns, baseline disease severity, and residual confounding.

In CAP-based cohorts, Wong et al. linked steatosis to advanced fibrosis at baseline [165]. In an analysis with 17 liver-related events, Uman et al. reported a positive association with fibrosis and an imprecise association with steatosis [166].

Huang R et al. [129] examined cause-specific mortality during antiviral treatment. Zhang et al. [49] associated MASLD with coronary heart disease (aHR 1.913, 95% CI 1.338–2.736); the corresponding estimate for overall cardiovascular disease was 1.160 (0.957–1.406). Their CHB population, as defined in the study, included HBsAg-negative participants. Yu et al. associated steatosis with incident type 2 diabetes in CHB and reported differences in glucose-metabolism associations according to previous viral-load trajectories and HBsAg seroclearance [167].

In the population-based HBsAg-positive Kailuan cohort, MASLD was associated with higher hazards of extrahepatic cancer and cardiovascular events. The estimate for primary liver cancer was imprecise and below 1 [168]. The number of eligible participants differed by outcome. Additional renal studies examined urinary tubular markers and preoperative comorbidity in patients selected for HCC care (Table S1D).

In the cross-sectional HBV-positive population studied by Lin et al. [50], NAFLD was associated with prevalent kidney disease as defined in the study (aOR 1.651, 95% CI 1.146–2.378; reference: HBV alone), after adjustment for age, sex, body mass index, diabetes and hypertension. HBV infection was defined by HBsAg positivity, with persistence unspecified. The kidney disease definition did not require abnormalities to persist for >3 months. Bogdan et al. [169] describe metabolic and inflammatory liver–kidney interactions in broader NAFLD populations, providing biological context. These processes differ from hepatorenal syndrome in advanced liver disease. The renal result concerns coexisting disease, whereas the cardiovascular cohorts assess subsequent events [49,168]. Hepatic outcomes and systemic disease can diverge: an apparent benefit at one endpoint can coexist with disease burden in other organs.

## 10. Clinical Implications and Cohort Design

### 10.1. Clinical Implications

Clinically, fibrosis and metabolic disease warrant assessment even when steatosis accompanies a favorable antigen-related or hepatic association. Antiviral treatment and HCC surveillance continue to follow established CHB guidance [3,4,5,6,153]; cardiovascular, renal, and alcohol-related assessment should reflect the patient’s risk profile.

### 10.2. Priorities for Cohort Design

To test the propositions in Section 3.3, prospective cohorts should measure hepatic fat, metabolic factors, and fibrosis in the same patients at comparable intervals. Repeated assessments should capture the duration and control of individual metabolic abnormalities, together with alcohol exposure, nutritional status, antiviral treatment, and surveillance; steatohepatitis should be characterized when available. Serial measurements are needed to establish whether fat loss precedes, accompanies, or follows fibrosis progression.

Common models should assess the incremental predictive value of metabolic history beyond current fat, fibrosis, and established CHB factors using calibration and discrimination. Prespecified comparisons by disease stage and viral suppression should account for baseline severity and treatment selection and report both relative associations and absolute risks. Antigen, fibrosis, HCC, decompensation, mortality, cardiovascular and renal outcomes should be followed together but defined separately. Diagnostic thresholds, analysis populations, reference groups and follow-up should be reported explicitly to make treatment studies comparable [25,48,170]. Table 3 summarizes the outcome-specific evidence.

## 11. Limitations

Evidence selection was purposive, and unavailable information left some potentially relevant comparisons unassessed (Supplementary Methods S1). Shared cohorts, including the Rotterdam and REAL-B series, and overlapping evidence syntheses limit independent replication.

Studies used different definitions of steatosis and metabolic disease and varied in the timing of measurements. Outcomes, reference groups and analyzed populations sometimes differed even within a report, complicating comparisons.

The evidence is predominantly observational and remains susceptible to selection bias, residual confounding, measurement error, changing exposures and differences in surveillance.

## 12. Conclusions

The CHB–SLD “friend or foe” debate combines questions with different prognostic meanings. Within-study comparisons show that inverse associations with current hepatic fat can coexist with adverse metabolic associations, and that favorable antigen-related findings can accompany fibrosis progression. Selected cohorts may also carry high absolute HCC risk despite inverse relative associations in a broader synthesis. Exposure definition, disease stage, patient selection, and outcome differences explain part of the apparent paradox; adverse steatosis findings and discrepancies in similar settings remain to be explained.

Observational evidence suggests that metabolic history and fibrosis stage may explain risk obscured by a current SLD label. Prospective cohorts should measure metabolic factors and hepatic fat repeatedly at comparable intervals to test whether metabolic factors add predictive value beyond hepatic fat and established CHB factors, and whether this contribution differs with viral suppression.

## Figures and Tables

**Table 1:** Selected comparisons of exposures, outcomes and clinical settings in chronic hepatitis B with steatotic liver disease.

Report and Comparison	Exposure and Reference Group	Outcome and Estimate (95% CI)	Analysis/Source	Study Details and Limitations
Huang SC 2023 [35] Exposure definition	Steatosis present versus absent; separately, each additional study-defined MAFLD metabolic condition.	HCC: aHR 0.45 (0.30–0.67) for steatosis; aHR 1.40 (1.19–1.66) per additional condition.	Multivariable Cox models; source Table 4.	Baseline count of source-defined MAFLD conditions. Related NTUH cohorts overlap.
Lin 2024 [36] Exposure definition	(i) MASLD versus absence of MASLD in the full CHB cohort; (ii) ≥3 versus <3 CMRFs within MASLD.	(i) HCC: aHR 3.996 (2.007–7.959) before IPTW; aHR 10.191 (4.327–24.002) after IPTW. (ii) CMRF-group cumulative HCC incidence: log-rank *p* = 0.066.	Multivariable Cox models before and after IPTW, with different selected covariates (source Table 2); CMRF groups: source Figures 2H and 3H.	36 HCC events overall. Classification and within-MASLD count analyses have different reference populations. The CMRF comparison is reported as a survival-curve test; a group-specific HR/CI is unavailable in the source.
Peleg 2019 [45] Exposure definition	Steatosis present versus absent; diabetes present versus absent; BMI per 1 kg/m^2^ increase.	Death-or-any-cancer composite: aHR 4.35 (1.69–8.99), 1.56 (0.83–2.99), and 0.97 (0.91–1.05), respectively.	Multivariable Cox model; source Table 3; 524 initially untreated patients.	Composite endpoint. Eligibility required repeated consistent ultrasound fat status; fat, diabetes and BMI estimates share the outcome and model.
Li J 2026 [46] Exposure definition	Diabetes present versus absent; moderate/severe versus mild histological steatosis.	Significant fibrosis (≥F2): aOR 1.86 (1.15–3.01) for diabetes; aOR 1.53 (1.04–2.24) for steatosis severity.	Multivariable logistic model after propensity-score matching; source Table 3.	Cross-sectional biopsy associations. Both diabetes and steatosis severity were associated with the same fibrosis endpoint.
Con 2024 [47] Exposure definition	Ultrasound steatosis present versus absent; separately, a source-defined binary metabolic composite incorporating overweight, diabetes, hypertension, and hyperlipidemia.	Model-estimated LSM trajectory effects at 8 years: 9.4% decrease with steatosis (95% CI, 1.3–16.8% decrease); +0.8% with the metabolic composite (95% CI, −6.1% to +8.4%).	General linear mixed-effects sensitivity model with time terms; Results, LSM section, p. 1499.	Relative percentage changes in a longitudinal surrogate. The metabolic variable is a composite exposure indicator. Fibrosis markers showed discordant patterns.
Huang SC 2025 [32] Exposure definition	(i) SLD present versus absent in the matched sample; (ii) MASLD versus non-MASLD within SLD.	All-cause mortality: (i) HR 0.62 (0.53–0.74); (ii) aHR 1.79 (1.24–2.58).	(i) Matched-sample survival analysis, source-labeled HR. (ii) Multivariable Cox model, source Figure 1A.	The analyses use different populations and reference groups. These estimates concern all-cause mortality; cause-specific analyses use separate models.
Mak 2020 [28] Outcomes and clinical setting	(i) Baseline CAP ≥ 248 versus <248 dB/m. (ii) Persistent severe steatosis versus persistent absence of steatosis in the descriptive follow-up comparison.	(i) HBsAg clearance: HR 3.246 (1.278–8.243). (ii) LSM-stage progression: crude proportions 41.3% versus 23.0%; *p* = 0.05.	(i) Cox analysis; baseline factors significant in univariate analysis entered the procedure (source Table 2). (ii) Unadjusted descriptive follow-up comparison (Results, p. 802).	Exposure timing and outcome differ between analyses. The displayed fibrosis percentages compare source-defined persistent groups and concern LSM-stage change over three years.
Chau 2025 [48] Outcomes and clinical setting	Imaging-defined SLD present versus absent; 868 matched pairs.	Adjusted SHR: virological response 0.97 (0.86–1.11); biochemical response 0.77 (0.68–0.88); combined response 0.84 (0.72–0.97).	Multivariable Fine–Gray models; source Table 4 and Table 2, and 3, respectively.	Virological response: HBV DNA <20 IU/mL. Biochemical response: sex-specific ALT normalization. Combined response is a composite. Imaging SLD retains the source definition; potential REAL-B overlap.
Zhang 2026 [49] Outcomes and clinical setting	MASLD present versus absent in the overall cohort of 8052 participants.	aHR: cirrhosis 0.606 (0.445–0.824); HCC 0.574 (0.410–0.804); coronary heart disease 1.913 (1.338–2.736); overall CVD 1.160 (0.957–1.406).	Overall-cohort Cox models adjusted for sex, age, BMI, hypertension, and diabetes; source Table 2.	Source-defined CHB population includes HBsAg-negative participants. Matching descriptions contain conflicting denominators; displayed estimates use the overall cohort. Coronary disease and overall CVD are separate endpoints.
Lin 2021 [50] HBV population	NAFLD within the HBV-positive population; reference: HBV alone.	Study-defined kidney disease: aOR 1.651 (1.146–2.378).	Source Table 4, model 3; logistic regression adjusted for age, sex, BMI, diabetes and hypertension.	Cross-sectional prevalence association in an HBsAg-positive population; HBV persistence was unspecified. The kidney-disease definition used eGFR, albuminuria, or proteinuria without a >3-month duration requirement.
Choi 2020 [30] Disease stage and changes over time	NASH and advanced fibrosis (AF, F3–F4) joint groups; common reference: NASH−/AF−.	Any clinical event: aHR 4.81 (2.59–8.95) for NASH+/AF+; aHR 2.30 (1.31–4.05) for NASH−/AF+. Within AF−, NASH groups: log-rank *p* = 0.248.	Cox model adjusted for age, sex, HBeAg and diabetes (source Table 3); within-stage survival comparison (source Figure 4). Source: NASH+/AF− adjusted estimate contains an internal inconsistency.	Composite: decompensation, HCC, transplantation or death. NASH grouping combines probable/definite NAS categories. Joint groups describe disease stage and phenotype. Related reports share a cohort source.
Yang B 2025 [51] Disease stage and changes over time	Persistent steatosis versus persistent absence of steatosis during nucleos(t)ide-analogue therapy.	Histological improvement: inflammation aOR 0.244 (0.090–0.665); fibrosis aOR 0.180 (0.064–0.507).	Multivariable logistic models, source Tables 3 and 4; source cohort of 219 patients with paired biopsies.	Lower odds of improvement. Baseline-injury subsets have different denominators; exposure trajectory and histological change are measured over the same follow-up period.
Wong YJ 2023 [39] Evidence synthesis	Fatty liver present versus absent in the weighted individual-patient-data analysis.	IPTW HR: HCC 0.68 (0.52–0.88); cirrhosis 0.61 (0.49–0.75); death 0.38 (0.25–0.57); HBsAg clearance 1.35 (1.14–1.60).	Individual-patient-data synthesis using inverse-probability weighting; source-labeled weighted HRs.	Component studies differ between aggregate and individual-data analyses. Ascertainment comparisons partly involve different cohorts. Component overlap creates dependence with original reports.
Mao 2023 [37] Evidence synthesis	Steatosis present versus absent; the same exposure contrast in the nucleos(t)ide-analogue-treated subgroup.	Pooled OR: HCC 1.59 (1.12–2.26); cirrhosis 1.52 (1.07–2.16); HBsAg clearance 2.22 (1.58–3.10). Treated-study HCC subgroup: OR 1.05 (0.62–1.77).	Study-level meta-analysis; source-reported pooled ORs. Primary effect measures and covariate adjustment varied.	Outcome and subgroup analyses involve different component studies and overlap with other syntheses. The treated subgroup is a separate study-level summary.

Parentheses following effect estimates contain 95% confidence intervals (CI). HR labels follow the original reports. Mak’s LSM-stage progression proportions are unadjusted; Con’s estimates are relative LSM changes. Table and figure numbers in the Analysis/Source column identify locations in the cited source reports. Abbreviations: AF, advanced fibrosis; ALT, alanine aminotransferase; BMI, body mass index; CAP, controlled attenuation parameter; CHB, chronic hepatitis B; CMRF, cardiometabolic risk factor; CVD, cardiovascular disease; eGFR, estimated glomerular filtration rate; HBeAg, hepatitis B e antigen; HBsAg, hepatitis B surface antigen; HBV, hepatitis B virus; HCC, hepatocellular carcinoma; HR, hazard ratio; OR, odds ratio; aHR/aOR, adjusted HR/OR; SHR, subdistribution HR; IPTW, inverse probability of treatment weighting; LSM, liver stiffness measurement; MAFLD, metabolic dysfunction-associated fatty liver disease; MASLD, metabolic dysfunction-associated steatotic liver disease; NAFLD, nonalcoholic fatty liver disease; NAS, NAFLD activity score; NASH, nonalcoholic steatohepatitis; SLD, steatotic liver disease; NTUH, National Taiwan University Hospital; REAL-B, Real-World Evidence from the Global Alliance for the Study of HBV.

**Table 2:** Steatotic and metabolic exposures, assessment methods, and outcomes in studies of chronic hepatitis B.

Exposure or Clinical Feature	Definition	Common Measurement or Ascertainment Methods	Limitations	Relevant Findings
Hepatic fat deposition	Describes the presence or amount of intrahepatic fat; metabolic dysfunction, steatohepatitis, and fibrosis-related injury require additional assessment [7,22].	Ultrasonography, CAP, MRI-PDFF, and histologic steatosis grading [7,116,117,118,119].	Primarily characterizes fat burden; limited ability to assess metabolic burden, inflammatory activity, hepatocellular injury, or fibrosis trajectory.	HBsAg seroclearance and fibrosis progression associated with CAP-defined steatosis [28].
NAFLD	Historical diagnostic label generally requiring steatosis after exclusion of significant alcohol use and other causes; operational thresholds varied across CHB cohorts.	Ultrasonography, CAP, biopsy, or clinical criteria used in the source study.	Alcohol thresholds, recall periods, and exclusion criteria vary; self-report may be incomplete, and older NAFLD cohorts differ from contemporary MASLD cohorts.	Baseline histologic features, fibrosis severity, and related outcomes reported in earlier NAFLD studies [120].
Alcohol exposure and MetALD	Captures combined metabolic and alcohol-related drivers; MetALD occupies an intermediate alcohol-exposure range between MASLD and alcohol-associated liver disease [22,26].	Current and lifetime alcohol intake in g/week; binge pattern; validated questionnaires such as AUDIT-C; alcohol biomarkers when appropriate [26].	Alcohol thresholds, ascertainment, and timing determine etiologic classification. CHB-specific outcomes under contemporary MetALD criteria require dedicated evaluation.	Direct CHB analyses concern measured alcohol exposure and metabolic syndrome; MetALD guidance supplies classification context [26,121].
MAFLD and MASLD	MAFLD: steatosis plus overweight/obesity, type 2 diabetes or ≥2 specified metabolic abnormalities. MASLD: steatosis plus ≥1 cardiometabolic risk factor and alcohol intake within the MASLD range. Both can coexist with CHB [20,21,22,23].	Clinical diagnostic criteria, imaging combined with metabolic measures, diabetes status, and records of other cardiometabolic risk factors.	Patients with the same diagnostic label may differ substantially in metabolic-risk composition and cumulative burden.	Significant fibrosis, cirrhosis and its complications, HCC, liver-related events, and mortality [24,31,32,33,34,35,122].
Histological steatohepatitis (NASH/MASH)	Steatosis with lobular inflammation and hepatocellular ballooning; retain the source diagnosis and stage fibrosis separately [22,116,118].	Liver biopsy or a source-study diagnosis of steatohepatitis.	Usually relies on biopsy; study populations may be selected, and fibrosis stage should be interpreted separately from steatohepatitis itself.	Baseline advanced fibrosis and later composite clinical events, within selected biopsy cohorts [30,123].
SLD identified from EHR codes	Broad diagnostic label in real-world databases that combines several ascertainment pathways and differs from imaging-based fat content or histologic injury.	ICD codes, diagnostic records, and healthcare databases.	Clinical ascertainment, code validation, testing frequency, and documentation determine the exposure represented by diagnostic codes.	Real-world associations with virologic outcomes, fibrosis, HCC, and mortality [29].
Metabolic exposure over time	Baseline risk-factor count, incident metabolic disease, and repeated glycemic measurements describe distinct exposure time scales.	Baseline metabolic criteria; repeated diabetes status, HbA1c, BMI, blood pressure, lipids, and weight.	Interpretation requires measurement timing, duration, and individual components; longitudinal trajectories require repeated observations.	Baseline counts relate to mortality and cirrhotic outcomes [32,33]; incident metabolic syndrome to LSM change [124]; glycemic measures to hepatic events [122].
Possible loss of liver fat in advanced disease	Other advanced liver-disease settings and cross-sectional CHB stage comparisons motivate a temporal fat-loss hypothesis [115,125,126].	Histologic or imaging assessment in advanced fibrosis or cirrhosis, interpreted together with disease stage and prior metabolic context.	Serial CHB fat, fibrosis and nutritional measurements are required to establish sequence and assess this explanation.	Hypothesis about exposure history and reverse causation.

Abbreviations: AUDIT-C, Alcohol Use Disorders Identification Test–Consumption; BMI, body mass index; CAP, controlled attenuation parameter; CHB, chronic hepatitis B; EHR, electronic health record; HbA1c, glycated hemoglobin; HBsAg, hepatitis B surface antigen; HCC, hepatocellular carcinoma; ICD, International Classification of Diseases; LSM, liver stiffness measurement; MAFLD, metabolic dysfunction-associated fatty liver disease; MASH, metabolic dysfunction-associated steatohepatitis; MASLD, metabolic dysfunction-associated steatotic liver disease; MetALD, metabolic dysfunction and alcohol-associated liver disease; MRI-PDFF, magnetic resonance imaging–proton density fat fraction; NAFLD, nonalcoholic fatty liver disease; NASH, nonalcoholic steatohepatitis; SLD, steatotic liver disease.

**Table 3:** Steatotic liver disease, metabolic factors and clinical outcomes in chronic hepatitis B.

Outcome	Main Interpretation	Exposure or Risk Factor	Representative Studies
Virologic and serologic outcomes	CAP-defined steatosis, clinically defined MASLD, and EHR-defined SLD have shown favorable antigen-related associations in some cohorts; a related quiescent-CHB cohort reported an inconclusive HBsAg-loss association. Confirmed seroclearance, seroconversion, and recorded antigen detection are distinct endpoints. Fibrosis trajectories require separate assessment.	CAP-defined steatosis, clinically defined MASLD, and EHR code-defined SLD.	Mak et al. [28]; Huang et al. [27]; Yendewa et al. [29]; Chang et al. [130]
Baseline fibrosis severity	Biopsy cohorts report inverse fat associations, greater injury with steatohepatitis or fat severity, and imprecise differences. These describe disease severity at biopsy.	Source-defined NAFLD, histological steatohepatitis, fat severity, diabetes, and metabolic count.	Yao et al. [120]; Li et al. [46,137]; Khalili et al. [123]; Gezer et al. [144].
Fibrosis progression and histologic injury	Repeated steatosis and metabolic measurements have been associated with LSM change or paired-biopsy improvement. Baseline injury, measurement method, and exposure timing differ. NASH with advanced fibrosis has also been associated with later composite events.	Persistent severe CAP-defined steatosis, MASLD, CMRF count, NASH and its histologic activity, T2D, and sustained glycemic burden.	Mak et al. [28,122]; Choi et al. [30]; Wong et al. [124]; Yang et al. [51]
HCC	HCC associations vary with exposure definition, metabolic factors, baseline stage, recruitment setting, and treatment. One database study found that the SLD–HCC association differed between men and women.	Steatosis, MASLD, metabolic count, joint fat/metabolic groups, fibrosis, treatment, and sex.	Mao et al. [37]; Wong et al. [39]; Huang et al. [35]; Adalı et al. [34]; Lin et al. [36]; Wang et al. [38]; Wu et al. [160]
Cirrhosis, decompensation, and liver-related events	Pre-existing metabolic dysfunction, metabolic risk-factor count, and incident diabetes have been associated with increased risks of cirrhosis or cirrhotic complications. MAFLD has also been associated with a higher risk of composite liver-related clinical events and death.	Pre-existing or incident metabolic dysfunction, CMRF count, incident diabetes, and MAFLD.	Huang et al. [33]; van Kleef et al. [24]
Mortality and cardiovascular outcomes	Reports describe inverse SLD–mortality associations and adverse fat or metabolic associations. Coronary disease and overall CVD have separate estimates and reference populations.	Steatosis, SLD/MASLD, CMRF count, and broader metabolic disease burden.	Huang SC et al. [32]; Huang R et al. [129]; Zhang et al. [49]; Peleg et al. [45]
Treatment response	Biochemical, virological, and antigen outcomes have distinct definitions and observed associations; combined response jointly requires its component outcomes.	Imaging-defined SLD; NAFL/NASH; steatosis trajectories.	Chau et al. [48]; Yang et al. [51]; Li et al. [131]; Chen et al. [132]
Renal outcomes	NAFLD was associated with prevalent kidney disease in an HBV-positive population. HBV persistence and kidney-disease duration were unspecified. General SLD studies provide background on possible mechanisms.	Source-defined NAFLD.	Lin et al. [50]; Bogdan et al. [169]

Abbreviations: CAP, controlled attenuation parameter; CHB, chronic hepatitis B; CMRF, cardiometabolic risk factor; CVD, cardiovascular disease; EHR, electronic health record; HBsAg, hepatitis B surface antigen; HBV, hepatitis B virus; HCC, hepatocellular carcinoma; LSM, liver stiffness measurement; MAFLD, metabolic dysfunction-associated fatty liver disease; MASLD, metabolic dysfunction-associated steatotic liver disease; NAFL, nonalcoholic fatty liver; NAFLD, nonalcoholic fatty liver disease; NASH, nonalcoholic steatohepatitis; SLD, steatotic liver disease; T2D, type 2 diabetes.

## Data Availability

This review generated no new primary research data. The source literature and evidence summaries are provided in the references and Supplementary Methods S1, Figure S1 and Tables S1 and S2.

## Supplementary Materials

The following supporting information can be downloaded at: https://www.mdpi.com/article/10.3390/medicina62101992/s1. Supplementary Methods S1: Literature Search and Selection; Figure S1: Literature identification, assessment and use; Table S1: Report Uses, Main Findings and Selection Reasons; Table S2: Five Examples of Supporting Literature and Their Uses. References [171,172,173,174,175,176,177,178,179,180,181,182,183,184,185,186,187,188,189,190,191,192,193,194,195,196,197,198,199,200,201,202,203,204,205,206,207,208,209,210,211,212,213,214,215,216,217,218,219,220,221,222,223,224,225,226,227,228,229,230,231,232,233,234,235,236,237,238,239,240,241,242,243,244,245,246,247,248,249,250,251,252,253,254,255,256,257,258,259,260,261,262,263,264,265,266,267,268,269,270,271,272,273,274,275,276,277,278,279,280,281,282,283,284,285,286,287,288,289,290,291,292,293,294,295,296,297,298,299,300,301,302] are cited in the Supplementary Materials.
