# Supplementary material for: Why Outcomes Diverge in Chronic Hepatitis B with Steatotic Liver Disease: Lessons from Within-Study Contrasts
Source: Medicina (Kaunas). 2026 Oct 15;62(10):1992. doi: 10.3390/medicina62101992 (PMC13706878; doi:10.3390/medicina62101992)
Supplement: Supplementary file 1 [file medicina-62-01992-s001.zip › medicina-4569625-supplementary.pdf]

Supplementary Materials

Why Outcomes Diverge in Chronic Hepatitis B with Steatotic Liver Disease: Lessons from Within-Study Contrasts

Supplementary Methods S1. Literature Search and Selection

The search covered publications available through 21 September 2026, without language or publication-type restrictions. PubMed covered all searchable years; Web of Science Core Collection covered Science Citation Index Expanded (SCI-EXPANDED) from 1992, Current Chemical Reactions (CCR-EXPANDED) from 1985, and Index Chemicus (IC) from 1993. PubMed used Medical Subject Headings (MeSH) and title/abstract fields; Web of Science used Topic fields and truncation. The full strategies are given below.

PubMed

```
("Hepatitis B, Chronic"[Mesh] OR "hepatitis B"[tiab] OR HBV[tiab] OR CHB[tiab]) AND ("Fatty Liver"[Mesh]
OR "Non-alcoholic Fatty Liver Disease"[Mesh] OR steatosis[tiab] OR steatotic[tiab] OR
steatohepatitis[tiab] OR "fatty liver"[tiab] OR NAFLD[tiab] OR NASH[tiab] OR MAFLD[tiab] OR MASLD[tiab]
OR MASH[tiab] OR SLD[tiab] OR MetALD[tiab] OR "metabolic dysfunction"[tiab] OR "metabolic
dysfunctions"[tiab] OR "metabolic syndrome"[tiab] OR "metabolic burden"[tiab] OR "metabolic risk"[tiab]
OR cardiometabolic[tiab] OR diabetes[tiab] OR diabetic[tiab] OR glycemic[tiab] OR glycaemic[tiab] OR
HbA1c[tiab] OR obesity[tiab] OR obese[tiab] OR overweight[tiab] OR adiposity[tiab] OR dyslipid*[tiab] OR
hyperlipid*[tiab] OR hypertension[tiab] OR "insulin resistance"[tiab])
```

Web of Science Core Collection

```
TS=("hepatitis B" OR HBV OR CHB) AND TS=(steatosis OR steatotic OR steatohepatitis OR "fatty liver" OR
NAFLD OR NASH OR MAFLD OR MASLD OR MASH OR SLD OR MetALD OR "metabolic dysfunction*" OR "metabolic
syndrome" OR "metabolic burden" OR "metabolic risk" OR cardiometabolic OR diabet* OR glycemic OR
glycaemic OR HbA1c OR obes* OR overweight OR adiposity OR dyslipid* OR hyperlipid* OR hypertension OR
"insulin resistance")
```

The manuscript references, existing revision bibliography, and all 46 references in Huang and Tseng (2026) [40] were also checked, adding 39 records: 35 scholarly records and 4 World Health Organization background documents.

Screening and Use of Evidence

Duplicate records were merged by PubMed identifiers, digital object identifiers (DOIs), and publication details. Titles, abstracts and existing assessments were reviewed first; relevant sections of the original reports were consulted when information was unclear. Additional information was checked for 945 records. Eligibility followed the population and comparison criteria in Section 2.3; the results are shown in Figure S1.

The databases yielded 14,424 records, reduced to 9,308 after deduplication and increased to 9,347 after supplementary identification. Of 258 reports meeting the comparison criteria, 119 contributed to outcome synthesis, including 14 detailed examples in Table 1. Figure S1 gives the counts for each branch; Table S1 records individual uses and selection reasons.

Cohort overlap was assessed from study centers, recruitment periods, participants, and follow-up. Different analyses of the same population were retained with their relationship noted. When a conference abstract and journal report described the same analysis, the journal report was used.

The 223 records with unresolved eligibility did not enter outcome synthesis. Links between 99 conference records and journal reports remained unconfirmed; 95 were also among the eligibility-unresolved records. These 99 records supplied no independent outcome evidence. A separate conference abstract used in Table S1D is identified as preliminary evidence.

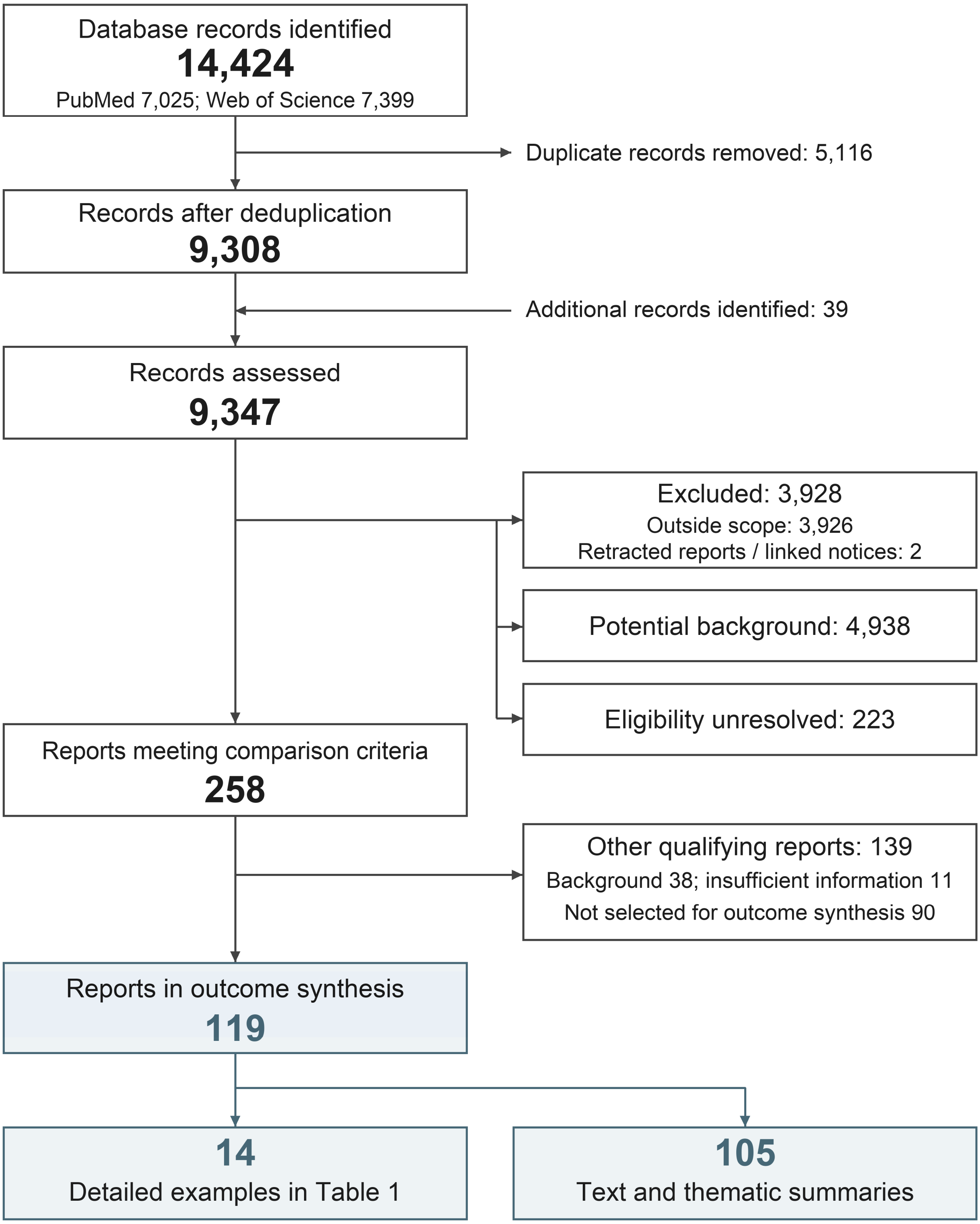

Figure S1. Literature identification, assessment and use. Assessment branches show the recorded classifications as of 25 September 2026; the 945 additional information checks occurred within this assessment. The 119 outcome reports comprise 14 Table 1 examples and 105 reports used in the text or thematic synthesis.

Supplementary Table S1. Report Uses, Main Findings and Selection Reasons

S1A–B contain 168 reports: 119 used in outcome synthesis, 38 for background, and 11 not used because of insufficient information for interpretation. S1E lists the other 90 reports that met the comparison criteria but were not selected. S1C explains the selection of the 14 examples; S1D summarizes the main findings by outcome.

Numbers in square brackets refer to the main manuscript bibliography; REC denotes the search record identifier. Abbreviations used in Tables S1 and S2 are defined at the end of this supplement.

The source-relationship column identifies known or possible population overlap and related publication versions; "—" indicates that no specific link was recorded. An "inconclusive association" means that the available results do not establish a clear association.

Table S1A. Original Studies and Their Uses (158 Reports, Including 1 Conference Abstract)

Report/use	Comparison and main findings	Cohort or source relationship
Chau et al. 2025 [111] REC00018 · Narrative discussion / Table 1	Matched nucleos(t)ide analogue-treated cohort: imaging-defined SLD was compared for viral suppression, ALT normalization, and composite response, with analyses by baseline viral and biochemical status.	Shares the REAL-B network with Huang R 2025 and possibly the Stanford source with Li 2020. Conference report REC07237 may share its source, with a different sample or analysis. Patient overlap remains unquantified.
Huang et al. 2025 [32] REC00019 · Narrative discussion / Table 1	All-cause and cause-specific mortality were compared by SLD, MASLD within SLD, cardiometabolic risk-factor counts and joint fat–metabolic groups.	Shares the NTUH 2006–2021 database with the Huang SC series; patient overlap is unquantified.
Yilmaz et al. 2015 [138] REC00031 · Narrative discussion	Cross-sectional biopsy comparisons linked steatosis to viral load, biochemical activity, necroinflammation, and fibrosis. Histological differences were imprecise; source details were limited.	—
Patmore et al. 2026 [68] REC00034 · Thematic synthesis	Five-year HCC risk varied by PAGE-B category in patients with metabolic comorbidity and the MASLD subset. All had metabolic comorbidity; cirrhotic patients retained residual risk in the low PAGE-B category.	Toronto/Rotterdam network intersection; patient overlap is unconfirmed.
Patmore et al. 2025 [145] REC00041 · Narrative discussion	MASLD and metabolic comorbidities were compared for liver stiffness and its trajectories before and after antiviral initiation, including MASLD-positive versus MASLD-negative groups.	Shared European/Rotterdam network. Conference report REC07034 may share its source, with a different sample or analysis. Patient overlap is unconfirmed.
Hu et al. 2024 [121] REC00047 · Thematic synthesis	Obese and non-obese CHB–NAFLD groups had inconclusive differences in three-year LSM-stage progression and virological/biochemical response. Full-cohort and post-treatment viral-suppression analyses used different subsets.	Wuhan Tongji source; possibly linked to other treatment reports. Patient overlap is unconfirmed.
Kalafateli et al. 2026 [148] REC00050 · Background	MASLD, BMI, and metabolic components were compared for stiffness-defined fibrosis and longitudinal stiffness in treated and untreated groups. Thresholds differed by group; denominators and estimates were inconsistent.	European multicenter network intersection; patient overlap is unconfirmed.

Report/use	Comparison and main findings	Cohort or source relationship
Xiong et al. 2024 [171]<br>DOI: 10.5152/tjg.2024.23512<br>REC00054 · Background	Biopsy inflammation and fibrosis were compared across attributed causes of persistent ALT elevation during treatment, including NAFLD. Diagnostic attribution and biopsy selection affect interpretation.	—
Tsai et al. 2025 [172]<br>DOI: 10.2174/0118715303323078241022050619<br>REC00055 · Not used: insufficient information	Incident metabolic syndrome was described in HBV–NAFLD/NASH groups, but subgroup counts were inconsistent. Adjusted hazards compared HBV status within fatty-liver strata rather than fatty liver within HBV.	—
Janczura et al. 2025 [84]<br>REC00061 · Thematic synthesis	European treated cohort: one-year HBV DNA negativity was 69.5% with SLD and 66.7% without. HBeAg seroconversion was similar, and ALT fell in both groups; only four HCC events occurred.	—
Choi et al. 2020 [30]<br>REC00077 · Narrative discussion / Table 1	Simple steatosis, steatohepatitis, and histological activity components were compared for liver-related events and mortality, including joint advanced-fibrosis–steatohepatitis groups.	Same Toronto (2005–2016) and Rotterdam (1985–2012) cohort as van Kleef 2021: 1,089 participants, of whom van Kleef excluded 13 for insufficient MAFLD information (n=1,076). Brouwer 2015 shares the Rotterdam source/period; overlap is unquantified. Conference report REC07605 uses the same cohort with updated analyses.
Li et al. 2026 [109]<br>REC00087 · Narrative discussion / Table 1	Diabetes, steatosis grade, metabolic syndrome and its components were compared for biopsy fibrosis; diabetes was also assessed for inflammation and steatosis severity.	Possible Nanjing biopsy-network overlap. Conference report REC07616 may share its source, with a different sample or analysis. Patient overlap is unconfirmed.
Li et al. 2025 [131]<br>REC00091 · Thematic synthesis	During pegylated-interferon add-on treatment, HBsAg-clearance differences were inconclusive, and ALT normalization was lower with NAFLD. ALT values follow source Table 3 because abstract labels conflict.	—
Hong et al. 2025 [31]<br>REC00096 · Narrative discussion	Histological fibrosis was compared by MASLD status and, within MASLD, cardiometabolic risk-factor counts and steatosis grades.	—
Yendewa et al. 2025 [29]<br>REC00097 · Narrative discussion	EHR-defined SLD was compared for hepatic events and mortality, with stratification by baseline cirrhosis.	Possible shared TriNetX source with REC00146; patient overlap is unquantified.
Zheng et al. 2013 [45]<br>REC00104 · Thematic synthesis	Cross-sectional biochemical, viral and histological comparisons: steatosis accompanied greater metabolic burden but less inflammation and fibrosis. Comparisons were unadjusted and subject to sample selection.	—

Report/use	Comparison and main findings	Cohort or source relationship
Huang et al. 2025 [33] REC00113 · Narrative discussion	Prevalent and incident metabolic abnormalities, risk-factor counts within SLD, and changing steatosis status were compared for cirrhosis and its complications.	Shares the NTUH 2006–2021 database with the Huang SC series; patient overlap is unquantified. Conference report REC07081 uses the same cohort with updated analyses.
Chang et al. 2022 [53] REC00117 · Thematic synthesis	Claims-defined NAFLD accompanied more elevated ALT. FIB-4 associations were imprecise for NAFLD and positive for diabetes; biochemical comparisons considered viral context.	—
Yao et al. 2024 [126] REC00118 · Narrative discussion	Untreated CHB: biopsy fibrosis was compared by NAFLD status and, within NAFLD, between mild and moderate-to-severe steatosis.	—
Wang et al. 2026 [168] REC00122 · Narrative discussion	HBV-positive participants: MASLD was compared for primary liver cancer, extrahepatic cancer, and cardiovascular disease, with analyses of fat severity, changing MASLD status, and ALT background.	—
Xiong et al. 2023 [173] DOI: 10.1097/md.0000000000033062 REC00123 · Background	HCC surgery patients: steatosis, MAFLD, and lean/nonlean MAFLD were compared for infectious and major postoperative complications against a common non-MAFLD reference.	Same center and intersecting years as REC01034/REC01176; overlap is unconfirmed.
Ceylan et al. 2016 [85] REC00125 · Thematic synthesis	Steatosis and steatohepatitis were compared for virological response; the overall association was inconclusive. The six-month TDF analysis included six steatohepatitis cases and was unadjusted, insufficient for regimen comparison.	—
Shiue et al. 2026 [170] REC00146 · Background	A small virally suppressed cohort compared CAP grades for stiffness and HBsAg; a separate TriNetX cohort assessed MASLD, cirrhosis, HCC, and survival. Endpoint inconsistencies limit event comparisons.	TriNetX component may overlap Yendewa 2025; extent unquantified.
Mak et al. 2021 [74] REC00155 · Thematic synthesis	Lower baseline CAP accompanied higher HCC risk; advanced fibrosis without severe steatosis had the highest incidence. Baseline phenotypes do not establish fat loss. Some subgroup models were logistic; untreated patients had four HCC events.	HKU CAP cohort; the initial 1,606 patients were reported by Seto 2018.
Con et al. 2024 [110] REC00161 · Narrative discussion / Table 1	MASLD, steatosis, and metabolic combinations were compared for liver-stiffness changes, with additional HBV DNA and ALT trajectories.	Conference report REC07447 uses the same cohort with updated analyses.
Khalili et al. 2021 [127] REC00183 · Narrative discussion	No fatty liver, simple steatosis, and steatohepatitis were compared for baseline biopsy fibrosis and subsequent biochemical and fibrosis-marker trajectories.	—

Report/use	Comparison and main findings	Cohort or source relationship
Han et al. 2026 [94]<br>REC00185 · Thematic synthesis	During Peg-IFN treatment, MASLD accompanied slower and lower complete virological response. HBsAg seroclearance was 15.4% versus 16.6%, with inconclusive differences. Follow-up LSM was higher without biopsy confirmation of progression.	—
Pokorska-Śpiewak et al. 2017 [174]<br>DOI: 10.1097/md.0000000000005832<br>REC00196 · Background	Pediatric CHB: steatosis was compared for biochemical, viral, inflammatory, and fibrosis measures. Only four steatosis cases were present; regression orientation does not establish temporal or causal direction.	—
Lee et al. 2019 [71]<br>REC00206 · Thematic synthesis	The biopsy steatosis–HCC association was positive before weighting but imprecise after metabolic balancing and in the noncirrhotic subgroup; only 17 HCC events occurred.	Included in Wong 2023 aggregate synthesis, but not its five IPD cohorts.
Lin et al. 2021 [113]<br>REC00207 · Narrative discussion / Table 1	HBV-positive participants: NAFLD was compared for renal abnormalities with a defined reference and adjustment set; joint HBV–NAFLD analyses were also reported.	—
Zhang et al. 2023 [141]<br>REC00216 · Thematic synthesis	Paired biopsies before and after 78 weeks of entecavir-based therapy compared four fat trajectories for fibrosis regression in 239 patients. Ishak, Beijing P-I-R, and exposure classifications used the same endpoint biopsy.	Trials NCT03777969 and NCT02849132 are different from Tang's trial. Conference records REC07743 and REC07814 are candidate links. Possible overlap with other biopsy sources is unquantified.
Huang et al. 2023 [35]<br>REC00221 · Narrative discussion / Table 1	MAFLD, steatosis, and metabolic-condition counts were compared for HCC, with analyses by cirrhosis and antiviral-treatment status.	Shares the NTUH 2006–2021 database with the Huang SC series; patient overlap is unquantified.
Chen et al. 2024 [132]<br>REC00222 · Narrative discussion	No steatosis, simple steatosis, and NASH were compared for the same virological-response endpoint during nucleos(t)ide analogue treatment.	Possible overlap with the Nanjing Second Hospital 2017–2022 biopsy series; unconfirmed.
Jin et al. 2012 [80]<br>REC00226 · Thematic synthesis	During entecavir treatment, source Table 5 showed lower DNA-clearance and later ALT-normalization proportions with steatosis; HBeAg differences were inconclusive. Inconsistent P values and models preclude quantitative interpretation.	—
Huang et al. 2022 [136]<br>REC00240 · Narrative discussion	FLIP/SAF-defined NAFLD, NASH and histological components were compared for fibrosis, with NASH–fibrosis analyses by HBeAg, ALT and lean status.	—
Mak et al. 2020 [28]<br>REC00250 · Narrative discussion / Table 1	Baseline steatosis status and grade were compared for HBsAg clearance; persistent or changing severe steatosis was assessed for stiffness-stage progression, with joint HBV DNA–fat groups.	HKU CAP series, linked to Seto 2018 and Mak reports; included in Wong and Mao 2023 syntheses.

Report/use	Comparison and main findings	Cohort or source relationship
Wang et al. 2026 [38]<br>REC00253 · Narrative discussion	Treated CHB: SLD and cardiometabolic burden were assessed separately and jointly for HCC, including cirrhosis strata and fat–metabolic groups with a common reference.	—
Li et al. 2020 [133]<br>REC00259 · Thematic synthesis	In 555 treated patients, NAFLD was compared for complete viral suppression, biochemical response, and combined response. Adjusted associations were inconclusive; outcome definitions and regimens follow the source report.	Stanford 2000–2016 cohort may overlap REAL-B, including Chau 2025; extent unquantified. Timing of pre-treatment NAFLD diagnosis was unavailable.
Enomoto et al. 2016 [92]<br>REC00264 · Thematic synthesis	Untreated, biopsy-selected HBeAg-negative patients: steatosis and ferritin accompanied ALT elevation in the low-DNA stratum, and HBV DNA in the high-DNA stratum. An interaction was not established.	—
Cho et al. 2020 [158]<br>REC00267 · Narrative discussion	Virally suppressed, treated CHB: imaging-defined NAFLD was compared for incomplete biochemical response and subsequent HCC.	—
Rastogi et al. 2011 [46]<br>REC00269 · Thematic synthesis	Steatosis accompanied lower HBV DNA; biopsy activity and fibrosis differences were inconclusive. Inconsistent viral quantities prevented transcription of exact values.	—
Xu et al. 2020 [59]<br>REC00270 · Thematic synthesis	Within biopsy stages F0–F1, NAFLD and higher CAP accompanied higher LSM and more false-positive fibrosis classifications. Inconsistent subgroup counts limit numerical presentation.	—
Lv et al. 2023 [142]<br>REC00276 · Thematic synthesis	Stiffness-selected HBV–MAFLD biopsy sample: hypertension and diabetes were associated with fibrosis. Selection and table inconsistencies limit comparison with steatosis grade or of association strength.	—
Lv et al. 2021 [175]<br>DOI: 10.1038/s41598-020-80709-z<br>REC00282 · Background	CHB steatosis was compared for ALT and stiffness; baseline regression used CAP as the outcome. An intervention targeting steatosis reduction provided information on determinants and management.	—
Seto et al. 2018 [55]<br>REC00284 · Thematic synthesis	Any steatosis, severe steatosis, and continuous CAP were compared in treated and untreated groups. Severe steatosis accompanied stiffness-defined severe fibrosis in both groups; these were prevalent surrogate stages.	Confirmed Seto–Mak HKU CAP series; reports address different comparisons.
Tang et al. 2023 [140]<br>REC00305 · Thematic synthesis	Four biopsy-defined fat trajectories from baseline to week 104 were compared for viral, biochemical, and histological outcomes in 164 HBeAg-positive patients. Source Table S4, model 3, used persistent absence of fat as the reference for fibrosis improvement.	EFFORT trial NCT00962533, 24 Chinese centers, 2009–2012; different from Zhang 2023's trials. Possible overlap with other Chinese biopsy reports is unquantified.
Wong et al. 2020 [165]<br>REC00325 · Narrative discussion	Steatosis and metabolic factors were compared for baseline advanced fibrosis; steatosis was also assessed for subsequent hepatic and cardiovascular events, malignancy and mortality by treatment group.	Included in Wong and Mao syntheses.

Report/use	Comparison and main findings	Cohort or source relationship
Charatcharoenwitthaya et al. 2017 [139]<br>REC00327 · Thematic synthesis	Steatohepatitis was assessed for baseline METAVIR fibrosis in 256 patients and viral, biochemical, and combined responses in 112. The reference combined simple steatosis and no steatosis.	Siriraj Hospital, Thailand, 2010–2013. The 48-week treatment subset came from the baseline biopsy cohort; each outcome has its own denominator. Conference links remain unconfirmed.
Zhu et al. 2016 [88]<br>REC00333 · Thematic synthesis	NAFLD accompanied early virological differences and lower later ALT normalization; later viral/HBeAg differences were inconclusive. Higher HOMA-IR within NAFLD accompanied lower ALT normalization. Seroconversion denominators and percentages contain errors.	—
Yang et al. 2025 [114]<br>REC00371 · Narrative discussion / Table 1	Paired biopsies during therapy defined absent, incident, persistent, and resolved steatosis. Trajectories were compared for viral, biochemical, inflammatory, and fibrosis improvement, including baseline lesion subgroups.	Beijing Ditan Hospital paired-biopsy cohort, 2010–2021. Tang 2023 and Zhang 2023 report different registered trials; complete patient independence is unconfirmed.
Zheng et al. 2010 [51]<br>REC00372 · Thematic synthesis	HBeAg-negative biopsy population: BMI and cholesterol were associated with fibrosis stage, and insulin with inflammation. Conflicting model coefficients preclude quantitative comparison of fat and metabolic measures.	—
Brouwer et al. 2015 [151]<br>REC00390 · Narrative discussion	Among 531 biopsy patients, 491 had follow-up for death and HCC. Age-adjusted steatohepatitis intervals crossed 1. Alcohol exposure was permitted; the source definition of steatohepatitis is retained.	Erasmus Rotterdam, 1985–2012, matching the source/period in Choi 2020 and van Kleef 2021; patient overlap is unquantified.
Ren et al. 2025 [176]<br>DOI: 10.1186/s12985-025-02642-9<br>REC00408 · Background	MASLD status and severity were compared for prespecified on-treatment HBsAg decline, including HBeAg strata. The endpoint was concentration decline rather than clearance; table inconsistencies limit quantitative comparison.	—
Xu et al. 2026 [52]<br>REC00420 · Thematic synthesis	HBV–MAFLD biopsy population: higher BMI was associated with significant inflammation; HDL was also assessed. Coding and table problems limit quantitative interpretation.	—
Zhang et al. 2026 [112]<br>REC00428 · Narrative discussion / Table 1	MASLD was compared for HCC and cardiovascular outcomes, with stratification by viral background.	—
Gezer et al. 2026 [144]<br>REC00439 · Narrative discussion	MASLD was compared for significant biopsy fibrosis; within MASLD, groups with one, two, or at least three cardiometabolic risk factors were compared for the same endpoint.	—
Ma et al. 2026 [177]<br>DOI: 10.1186/s12876-025-04488-5<br>REC00443 · Background	Selected noncirrhotic CHB: NAFLD was compared for colonoscopy-detected polyps. CAP, BMI, diabetes, and lipids were assessed within CHB–NAFLD in cross-sectional screening analyses.	—

Report/use	Comparison and main findings	Cohort or source relationship
Gu et al. 2026 [178]<br>DOI: 10.1186/s12876-025-04594-4<br>REC00446 · Background	Treated CHB–NAFLD: a diagnostic model assessed BMI and HDL for prevalent stiffness-defined significant fibrosis; the HDL association was inconclusive.	—
Liang et al. 2026 [97]<br>REC00463 · Thematic synthesis	Peg-IFN treatment: HBsAg-clearance and virological differences were inconclusive, while biochemical response was lower with NAFLD. The NAFLD×HBeAg interaction was inconclusive; ALT percentages were uncertain.	—
Dai et al. 2023 [49]<br>REC00531 · Thematic synthesis	Across untreated HBV phases, fatty liver accompanied biopsy inflammation. Fibrosis associations varied by endpoint and phase; formal interaction evidence did not establish phase modification.	Single-center Zhejiang biopsy source, 2016–2021; no specific cross-report link identified.
Huang et al. 2024 [27]<br>REC00544 · Narrative discussion	Steatosis, MASLD and cardiometabolic risk-factor counts were compared for HBsAg clearance, with stratification by fat severity and baseline HBsAg level.	Shares the NTUH 2006–2021 database with the Huang SC series; patient overlap is unquantified.
Shen et al. 2015 [179]<br>DOI: 10.1186/s12876-015-0263-9<br>REC00546 · Background	Small cross-sectional groups: alcoholic and nonalcoholic fatty liver were compared for prevalent diabetes against no steatosis. The nonalcoholic-fatty-liver association was inconclusive; triglycerides were also assessed.	—
Makuza et al. 2022 [180]<br>DOI: 10.3390/v14112579<br>REC00561 · Background	Administrative data compared liver-related mortality across joint HBV–NAFLD groups. Group-specific rates supported descriptive within-HBV comparisons; models used an uninfected reference.	British Columbia Hepatitis Testers Cohort, linked to other British Columbia reports.
Li et al. 2021 [75]<br>REC00571 · Thematic synthesis	Fatty liver accompanied lower cirrhosis/HCC hazards in matched treated patients; HBsAg-clearance intervals crossed 1 in treated and untreated groups. Methods specified univariable Cox models after matching, while Results used “adjusted” labels; associations were observational.	Included in Wong 2023 IPD analysis and Mao 2023 synthesis.
Kim et al. 2019 [81]<br>REC00604 · Thematic synthesis	During nucleos(t)ide analogue treatment, higher CAP accompanied lower probabilities of virological response and HBeAg loss. HCC associations were inconclusive with 25 events; HBeAg analyses used the positive subset.	Severance/Yonsei, 2007–2016: same center and intersecting years as Chang 2021 (REC01092); patient overlap is unconfirmed. Conference report REC07691 uses the same cohort with updated analyses.
Enomoto et al. 2020 [96]<br>REC00622 · Thematic synthesis	In the 64-person Peg-IFN response subset, fat and metabolic comparisons were inconclusive. Response required HBV DNA <2000 IU/mL and HBeAg negativity; genetic analyses addressed a separate question.	Linked to two Japanese sequential-treatment reports; overlapping patient count unverified.

Report/use	Comparison and main findings	Cohort or source relationship
Chen et al. 2020 [181]<br>DOI: 10.1186/s12876-020-01289-w<br>REC00628 · Not used: insufficient information	Steatosis grades were compared for HBeAg clearance and viral response within nucleos(t)ide analogue regimens. Reversed group labels and unclear supplementary strata prevent reliable extraction of direction.	—
Cheng et al. 2013 [182]<br>DOI: 10.1371/journal.pone.0072049<br>REC00635 · Background	HBV-positive fatty-liver health-check subset: waist circumference and HDL were associated with ALT elevation in cross-sectional metabolic comparisons.	—
Atalay et al. 2022 [86]<br>REC00744 · Thematic synthesis	Steatosis grades and steatohepatitis were compared for nucleos(t)ide analogue response; overall associations were inconclusive. Ordinal-model coefficients were not HRs/ORs; response windows and tables were inconsistent.	—
Toh et al. 2026 [157]<br>REC00748 · Narrative discussion	Steatosis, MASLD and cardiometabolic factors were compared for HCC, including joint MASLD–aMAP groups and an analysis excluding baseline cirrhosis.	—
Dong et al. 2015 [61]<br>REC00874 · Thematic synthesis	Biopsy-referenced comparisons: steatosis associations with ARFI, FibroScan and the Forns index were inconclusive. Inflammation and tissue fibrosis related more clearly to these measures.	—
Yi et al. 2024 [183]<br>DOI: 10.1186/s12985-023-02277-8<br>REC00926 · Background	MAFLD and steatosis grades were compared for pgRNA, HBV DNA, HBsAg, and HBeAg by HBV-DNA stratum in cross-sectional concentration and detectability analyses.	—
Cai et al. 2025 [135]<br>REC00969 · Narrative discussion	Normal-ALT, biopsy-selected CHB: MASLD was compared for inflammation, fibrosis and one-year DNA clearance, including BMI/lipid models, viral/injury strata and interaction analyses.	—
Natour et al. 2025 [184]<br>DOI: 10.1111/jvh.70071<br>REC01016 · Background	MASLD and diabetes were compared against the same shear-wave-elastography fibrosis threshold, alongside FIB-4 performance, in diagnostic analyses by metabolic group.	—
Xiong et al. 2025 [185]<br>DOI: 10.1038/s41598-025-03244-9<br>REC01034 · Background	MAFLD and diabetic/nondiabetic MAFLD subtypes were compared for recurrence-free and overall survival after HCC surgery; diabetes was also analyzed within CHB–MAFLD.	Probably near-nested with REC01176, from the same center/period; periods also intersect REC00123.

Report/use	Comparison and main findings	Cohort or source relationship
Mak et al. 2023 [120]<br>REC01045 · Narrative discussion	CAP, diabetes duration, mean HbA1c, and time to glycemic control were compared for HCC and stiffness-stage progression.	Confirmed HKU CAP series; patient overlap across Mak/Seto reports is incompletely quantified.
Wang et al. 2026 [125]<br>REC01055 · Narrative discussion	HBV MRI analyses related fat grade to corrected T1 and compared PDFF and corrected T1 by cirrhosis status. Cross-sectional stage comparisons do not establish within-person fat loss.	Voluntary ELEGANCE imaging substudy; participants are included in the parent cohort.
Amano et al. 2025 [69]<br>REC01070 · Thematic synthesis	During viral suppression, MAFLD accompanied HCC in logistic analyses, with differences across PAGE-B strata. MAFLD was assessed at final observation or HCC diagnosis, limiting prospective interpretation.	—
Chang et al. 2021 [130]<br>REC01092 · Narrative discussion	In 720 patients with quiescent CHB, CAP-defined steatosis was compared for HBsAg loss, stiffness-stage progression and HCC. Steatosis coefficients were univariate and inconclusive; HBsAg loss required one negative result.	Severance Hospital, Korea, 2004–2015: same center and intersecting years as Kim 2019 (REC00604); patient overlap is unconfirmed. Different from Mak's Hong Kong source.
Huang et al. 2025 [129]<br>REC01104 · Narrative discussion	During nucleos(t)ide analogue treatment, metabolic-disease counts, diabetes and steatosis were compared for cirrhosis, HCC and cause-specific mortality, including clinical strata.	Shares the REAL-B network with REC00018; patient overlap is unquantified.
Lee et al. 2016 [72]<br>REC01129 · Thematic synthesis	Entecavir-treated cohort: adiposity and steatosis had imprecise HCC estimates, while cirrhosis was associated with HCC; only seven events occurred.	—
van Kleef et al. 2021 [24]<br>REC01150 · Narrative discussion	MAFLD, steatohepatitis/NAS within MAFLD, and steatosis without metabolic risk were compared for clinical events.	Same Toronto (2005–2016) and Rotterdam (1985–2012) cohort as Choi 2020: 1,089 participants, with 13 excluded for insufficient MAFLD information (n=1,076). Brouwer 2015 shares the Rotterdam source/period; patient overlap is unquantified.
Xiong et al. 2024 [186]<br>DOI: 10.1186/s12957-024-03385-7<br>REC01176 · Background	HCC surgery population: MAFLD and three metabolic phenotypes within CHB–MAFLD were compared for overall, infectious, and major postoperative complications.	Probably near-nested with REC01034; center/period intersect REC00123.
Jang et al. 2024 [187]<br>DOI: 10.1002/kjm2.12781<br>REC01230 · Background	HBV stratum of a mixed-viral community air-pollution study: MAFLD and BMI were assessed for FibroScan-defined advanced fibrosis in univariable and multivariable analyses.	—
Gao et al. 2025 [101]<br>REC01257 · Thematic synthesis	Fatty-liver proportions across normal/abnormal urinary β2-microglobulin were 22.3%/25.5% in treated and 28.6%/40.5% in untreated patients. Both differences were inconclusive; these were cross-sectional renal tubular-marker comparisons.	—

Report/use	Comparison and main findings	Cohort or source relationship
Jiang et al. 2021 [188]<br>DOI: 10.1042/bsr20211769<br>REC01289 · Background	Joint HBV–fatty-liver groups were compared for contemporaneous ALT, AST, GGT, and lipid abnormalities against a common HBV-negative reference.	—
Cao et al. 2025 [189]<br>DOI: 10.1186/s12876-025-03646-z<br>REC01350 · Not used: insufficient information	Age-matched CHB, CHB–MASLD and MASLD-only groups were compared for biopsy inflammation and fibrosis. Repeated reports, definitions, and denominators were unclear.	—
Villa et al. 2022 [190]<br>DOI: 10.1002/hep4.2000<br>REC01448 · Background	HBsAg-positive participants: CAP and metabolic measures were compared for stiffness. All had HIV coinfection and antiretroviral exposure.	—
Alshuwaykh et al. 2022 [191]<br>DOI: 10.1002/hep4.2064<br>REC01527 · Not used: insufficient information	NAFLD–HCC comparisons included untreated patients outside treatment criteria and compensated cirrhosis with undetectable DNA. Coding and table-row inconsistencies prevent reliable extraction of direction.	—
Liu et al. 2025 [192]<br>DOI: 10.1177/03000605251379253<br>REC01774 · Not used: insufficient information	Fatty liver, BMI, glucose, triglycerides, and diabetes were compared for significant fibrosis. Conflicting endpoint definitions and table misalignment leave association directions unclear.	—
Adali et al. 2024 [34]<br>REC01831 · Narrative discussion	Untreated CHB: MASLD, steatosis and metabolic risk-factor counts were compared for HCC; adjustment status follows each source table.	—
Huang et al. 2021 [143]<br>REC01836 · Narrative discussion	Within HBV, MAFLD, NAFLD, and steatosis without metabolic risk were compared for significant inflammation and advanced fibrosis, including inconclusive histological differences.	—
Shao-Wen Wang et al. (2024) [193]<br>DOI: 10.4254/wjh.v16.i12.1429<br>REC01962 · Background	HBV-positive participants: MASLD was compared for FIB-4, ALT, and carotid plaque as cross-sectional surrogate measures rather than incident clinical events.	—
Ye et al. 2019 [194]<br>DOI: 10.1186/s13098-019-0440-z<br>REC01981 · Background	CHB–NAFLD: obesity and hypertension were assessed for insulin resistance; HOMA-IR quantiles were compared for metabolic abnormalities and ALT elevation in cross-sectional analyses.	—

Report/use	Comparison and main findings	Cohort or source relationship
Abu Baker et al. 2024 [77] REC01982 · Thematic synthesis	Cirrhosis was more frequent with MASLD, but the adjusted interval crossed 1; diabetes remained associated. HCC rates were similar; seroconversion denominators were small.	—
Yu et al. 2022 [156] REC01993 · Narrative discussion	Long-term cohort: steatosis, MAFLD, diabetes and obesity were assessed for HCC, with additional HBsAg-clearance and mortality analyses.	GECC civil-servant source, linked to Hsueh 2022 (REC02537). Main-cohort, case-cohort and extended-follow-up reports are linked; patient overlap is unquantified.
Yang et al. 2024 [102] REC02016 · Background	HCC population: preoperative cardiovascular disease, kidney disease, cirrhosis, and portal hypertension were compared by MAFLD. Extrahepatic differences were inconclusive; all comparisons concerned comorbidity prevalence.	Guangxi Cancer Hospital, intersecting periods; possible overlap with REC02014/REC02223.
Cheng et al. 2023 [195] DOI: 10.1016/j.jhepr.2023.100836 REC02020 · Background	HBV population: MAFLD was compared for FIB-4, NFS, carotid intima-media thickness, and plaque. Fibrosis-score directions differed, and plaque differences were inconclusive; all were cross-sectional surrogates.	—
Rugivarodom et al. 2023 [152] REC02038 · Narrative discussion	Biopsy steatosis categories and metabolic factors were compared for death/transplantation and hepatic events, including lower-fibrosis-stage subgroups.	—
Huynh et al. 2024 [196] DOI: 10.62347/ljrg3048 REC02059 · Background	MASLD and metabolic measures were compared for recurrence and survival after HCC resection, including causes of death and sex, BMI, and cirrhosis subgroups.	—
Yun et al. 2022 [155] REC02096 · Narrative discussion	Among 197,346 Korean NHIS participants with CHB, FLI-defined MAFLD, metabolic subtypes and repeated MAFLD status were compared for HCC and mortality; non-MAFLD was the main reference.	Korean NHIS, 2010–2018; baseline health examinations in 2011–2012. Repeated-status analyses share the main cohort. Possible overlap with other national insurance studies is unquantified.
Wang et al. 2022 [76] REC02119 · Thematic synthesis	MAFLD and diabetes accompanied higher incident cirrhosis risk, including treatment strata. Follow-up began at first presentation, while MAFLD combined baseline and follow-up diagnoses, limiting temporal interpretation.	—
Lin et al. 2024 [36] REC02145 · Narrative discussion / Table 1	MASLD, NAFLD reclassification and metabolic-factor counts were compared for HCC, with stratification by advanced fibrosis and antiviral treatment.	—
Tan et al. 2023 [159] REC02163 · Not used: insufficient information	NAFLD, NASH, ballooning, and metabolic factors were compared for liver-related events; cirrhosis and HCC used different risk sets. Sparse HCC events and conflicting intervals limit numerical extraction.	—
Ko et al. 2022 [56] REC02175 · Thematic synthesis	Severe steatosis accompanied less detectable HBV DNA and more stiffness-defined fibrosis. Comparisons were cross-sectional, with heterogeneous treatment histories.	Canadian HBV Network; patient overlap with other network reports is unconfirmed.

Report/use	Comparison and main findings	Cohort or source relationship
Zhang et al. 2023 [82]<br>REC02208 · Thematic synthesis	Biopsy-defined NAFLD accompanied delayed first complete virological response, with longer estimated response times at higher fat grades. HBeAg and functional-cure differences were inconclusive; denominators varied by endpoint/time.	—
Wu et al. 2026 [17]<br>REC02212 · Background	The Rab7–STING mechanistic study also compared Sirius-red area in controls, MASLD, CHB, and CHB–MASLD. Human sampling details were incomplete; fibrosis comparisons were descriptive and the main contribution mechanistic.	—
Ko et al. 2025 [197]<br>DOI: 10.62347/ksln5850<br>REC02215 · Background	HCC surgical cohort: MASLD-plus-HBV and HBV-alone groups were compared for postoperative recurrence and mortality.	—
Liu et al. 2022 [198]<br>DOI: 10.3389/fonc.2021.783339<br>REC02223 · Background	After HCC surgery, CHB with/without MAFLD was compared for overall and recurrence-free survival, major perioperative complications, and 30-day mortality; complication differences were inconclusive.	Guangxi Cancer Hospital, intersecting periods; possible overlap with REC02014/REC02016.
Sharif et al. 2019 [65]<br>REC02237 · Thematic synthesis	Patients with mildly elevated ALT: steatosis and metabolic features accompanied ALT variation; HBV DNA associations with CAP and LSM were inconclusive. Exact source-table values require clarification.	—
Diao et al. 2022 [57]<br>REC02239 · Thematic synthesis	Normal-ALT CHB: severe steatosis accompanied LSM-defined significant fibrosis. Steatosis and metabolic syndrome were associated with ALT variation within the normal range.	—
Bodakci et al. 2026 [98]<br>REC02243 · Thematic synthesis	Higher biopsy steatosis grade accompanied HBsAg loss; follow-up LSM was higher with steatosis in both HBV phases. Paired histological progression was not shown; phase and treatment settings coincided.	—
Lin et al. 2021 [199]<br>DOI: 10.3390/jpm11080684<br>REC02250 · Background	After HCC surgery, MAFLD and diabetes were compared for recurrence and death/transplantation; lean status was assessed for recurrence within MAFLD.	—
Wang et al. 2025 [200]<br>DOI: 10.62347/uuck9838<br>REC02251 · Not used: insufficient information	Entecavir treatment: MAFLD was compared for viral, serological, and biochemical responses and post-treatment stiffness. Sex totals conflicted, denominators were unclear and FibroScan values were inconsistent.	—
Li et al. 2024 [83]<br>REC02256 · Thematic synthesis	NAFLD accompanied partial/delayed virological response to nucleos(t)ide analogues in both viral-load strata; timepoint labels conflicted.	—
Liu et al. 2026 [201]<br>DOI: 10.2147/jhc.s577506<br>REC02257 · Background	CHB-specific analysis after HCC surgery: SLD and diabetes were separately compared for all-cause mortality.	—

Report/use	Comparison and main findings	Cohort or source relationship
Wu et al. 2026 [160]<br>REC02259 · Narrative discussion	SLD–HCC associations were estimated in joint treatment-by-sex strata, with interaction testing.	—
Xue et al. 2022 [202]<br>DOI: 10.2147/cmar.s368366<br>REC02264 · Background	HCC patients: MAFLD, diabetes, and lipids were compared for progression/death; metabolic-burden groups within MAFLD shared a non-MAFLD reference.	—
Al-Busafi et al. 2024 [147]<br>REC02271 · Thematic synthesis	Inactive CHB: NAFLD accompanied more FIB-4 values >1.3. Few cases and a nonhistological threshold limit extrapolation.	—
Zhang et al. 2026 [203]<br>DOI: 10.1016/j.isci.2026.117362<br>REC02275 · Background	A natural killer-cell mechanistic study also compared clinical steatosis groups for ALT, bilirubin, fibrosis markers, and stiffness. Measurement timing and surrogate-defined endpoints limit prognostic interpretation.	—
Lim et al. 2020 [73]<br>REC02277 · Thematic synthesis	Biopsy-based HCC analysis: the univariable steatosis HR was >1, with an interval narrowly crossing 1; diabetes entered the adjusted model. Only 27 events occurred, and estimates were imprecise.	—
Peleg et al. 2019 [108]<br>REC02284 · Narrative discussion / Table 1	Steatosis, diabetes and BMI were compared for death/any cancer, with viral-load strata and joint steatosis–viral-load analyses.	Rabin Medical Center, 2007–2017; possible overlap with later Peleg reports, unquantified. Conference report REC07700 uses the same cohort with updated analyses.
Yun et al. 2022 [204]<br>DOI: 10.3390/cancers14205002<br>REC02290 · Background	HCC surgical cohort: metabolic proxy-defined MAFLD was compared for postoperative recurrence and all-cause mortality.	—
Hu et al. 2024 [66]<br>REC02291 · Thematic synthesis	CHB with steatosis: antiviral treatment accompanied less LSM-stage increase. Virological/biochemical response differences by steatosis grade were inconclusive; interaction was not established.	—
Santos et al. 2022 [205]<br>DOI: 10.4254/wjh.v14.i8.1652<br>REC02296 · Background	MAFLD and a body-shape index were compared for muscle-mass-to-BMI and handgrip strength. Cross-sectional timing, shared BMI components, and sample selection limit prognostic interpretation.	—
Gu et al. 2025 [87]<br>REC02297 · Thematic synthesis	Entecavir treatment: NAFLD accompanied milder baseline histology, similar early viral response, and lower 96-week DNA negativity. HBsAg concentration and clearance were reported separately; percentages were uncertain.	—
Li et al. 2026 [137]<br>REC02298 · Narrative discussion	Normal-ALT, biopsy-selected CHB: NAFLD groups were compared for inflammation and fibrosis, with composite liver-injury analyses by HBeAg and HBV DNA.	—

Report/use	Comparison and main findings	Cohort or source relationship
Chen et al. 2022 [50] REC02304 · Thematic synthesis	MAFLD was associated with inflammation and, in HBeAg-negative patients, fibrosis; HBeAg interaction tests were inconclusive.	—
Uman et al. 2025 [166] REC02306 · Narrative discussion	CAP-defined SLD was compared for biochemical, stiffness, and viral measures and long-term liver-related events; viral suppression was analyzed in treated and untreated groups.	—
Van Iderstine et al. 2021 [64] REC02321 · Thematic synthesis	After HBeAg-stratified matching, NAFLD accompanied lower FIB-4 in HBeAg-negative patients; FIB-4 and liver-enzyme differences were inconclusive in HBeAg-positive patients.	—
Sun et al. 2019 [54] REC02327 · Thematic synthesis	Abdominal obesity: NAFLD and metabolic components were compared for stiffness-defined fibrosis. The univariate NAFLD association was inconclusive; reference coding requires clarification.	—
Wu et al. 2013 [47] REC02350 · Thematic synthesis	Biopsy histological differences between steatosis groups were inconclusive; separate adiponectin analyses addressed mechanisms.	—
Chen et al. 2024 [146] REC02357 · Narrative discussion	MRI-PDFF and BMI were compared for liver stiffness, including within biopsy-defined fibrosis stages, alongside biochemical, viral and histological analyses.	—
Lin et al. 2019 [99] REC02365 · Thematic synthesis	Prediabetes/T2DM accompanied slower HBsAg decline; source-defined advanced fatty liver accompanied faster decline. Exposures were assessed across follow-up; its supplemental advanced-fat definition was unavailable in the assessed material.	—
Huang et al. 2025 [134] REC02369 · Narrative discussion	REAL-B fat–metabolic groups shared a reference for biochemical response. Additional comparisons assessed steatosis for HBsAg loss and MASLD for biochemical, virological, and composite responses.	REAL-B network, with recruitment features shared by REC00018 and REC01104; patient overlap is unquantified.
Nascimento et al. 2012 [48] REC02377 · Thematic synthesis	In the CHB subgroup, steatosis associations with pathological features and fibrosis were inconclusive. Only three steatosis cases were present; separate domain estimates were unavailable.	—
Luo et al. 2024 [67] REC02509 · Thematic synthesis	MRI-defined steatosis accompanied higher platelet counts and the absence of diameter-defined portal hypertension. Cross-sectional measurements did not track within-person fat loss.	—
Wu et al. 2024 [100] REC02510 · Thematic synthesis	Outcome-matched Peg-IFN cohort: steatosis-grade and ultrasound differences between cured/uncured groups were inconclusive; baseline CAP correlated inversely with HBsAg concentration. Experiments did not establish a causal clearance effect.	—
Hsueh et al. 2022 [70] REC02537 · Thematic synthesis	Steatosis accompanied greater HBsAg loss and lower HCC risk; PNPLA3 risk variants and obesity accompanied higher HCC risk.	GECC male civil-servant cohort, linked to Yu 2022 (REC01993); patient overlap is unquantified.

Report/use	Comparison and main findings	Cohort or source relationship
Fang et al. 2024 [63]<br>REC02569 · Thematic synthesis	Fatty liver accompanied higher ALT; LSM, FIB-4, and APRI differences were inconclusive overall and in most viral strata.	—
Liang et al. 2020 [95]<br>REC02644 · Thematic synthesis	Higher biopsy steatosis grade accompanied lower Peg-IFN virological, biochemical, and composite response at week 48 and 24 weeks after treatment. Follow-up included 206 of 226 patients; severe-steatosis subgroups were small.	—
Hao et al. 2025 [206]<br>DOI: 10.2147/jhc.s536629<br>REC02667 · Background	After HCC surgery, steatosis, BMI, diabetes and HDL were compared for recurrence-free survival, including tumor number/size, microvascular invasion and AFP groups, to inform a prediction model.	Qingdao resection cohort, 2010–2023; possible overlap with an earlier model cohort, unconfirmed.
Chen et al. 2017 [150]<br>REC02727 · Narrative discussion	Five-year follow-up compared steatosis and BMI for incident cirrhosis, and steatosis for APRI and virological response, including APRI analyses by treatment/virological-response group.	—
You et al. 2023 [207]<br>DOI: 10.2147/dmso.s416280<br>REC02790 · Background	HCC patients: MAFLD and metabolic components were compared for overall survival, including viral-background and metabolic-component subgroups.	—
He et al. 2020 [208]<br>DOI: 10.14218/jcth.2019.00053<br>REC02795 · Not used: insufficient information	Steatosis groups were compared for biochemistry, viral load, stiffness, and serum fibrosis indices. Apparently reversed CAP group labels leave cross-sectional association directions unclear.	—
Wang et al. 2026 [60]<br>REC02908 · Thematic synthesis	Low-ALT CHB: within biopsy stages S0–S1, moderate-to-severe steatosis accompanied higher LSM and more false-positive significant-fibrosis classifications; threshold labels require checking.	—
Dai et al. 2022 [209]<br>DOI: 10.3389/fonc.2022.811084<br>REC03102 · Background	After HCC resection, metabolic syndrome, BMI, and steatohepatitis were compared for overall and recurrence-free survival; steatohepatitis associations were inconclusive for both.	Seven-center surgical source including West China; links to other resection cohorts are unconfirmed.
Lak et al. 2025 [62]<br>REC03114 · Thematic synthesis	Small treated/untreated groups: CAP–LSM and BMI–LSM correlations were weak and inconclusive; source inconsistencies limit numerical presentation.	—
Li et al. 2026 [210]<br>DOI: 10.1016/j.jhepr.2026.101877<br>REC03247 · Background	HCC liver transplant recipients: no MASLD, simple MASLD, and MASH were compared for overall and recurrence-free survival; simple-MASLD differences were inconclusive.	Same 274 participants as REC02367, with shared controls and different exposure definitions. Overlap with other transplant reports is unresolved.
Al-Shuaili et al. 2024 [58]<br>REC03266 · Thematic synthesis	Inactive CHB: ultrasound fatty changes were associated with SWE-defined significant fibrosis after adjustment. The design was cross-sectional; values elsewhere in the source tables were inconsistent.	—

Report/use	Comparison and main findings	Cohort or source relationship
Ren et al. 2021 [211]<br>DOI: 10.21037/qims-19-1091<br>REC03740 · Not used: insufficient information	LiSA and CAP were correlated with concurrent stiffness and biochemical liver-injury measures. Incompatible coefficients and P values limit extraction, including of inconclusive biochemical findings.	—
Yoon et al. 2020 [161]<br>REC00156 · Narrative discussion	After HBV-HCC resection, histological NAFLD and NASH were compared for recurrence-free and overall survival. The unadjusted NAFLD survival advantage attenuated after adjustment; NASH differences were inconclusive.	Resection cohort, 2009–2013; patient overlap with other Korean reports is unconfirmed.
Wu et al. 2025 [162]<br>REC07050 · Narrative discussion	During immune checkpoint inhibition for HBV-HCC, MASLD was compared for progression-free and overall survival, contrasting weighted MASLD analyses with individual metabolic factors.	Chinese multicenter cohort, October 2017–April 2023; patient overlap with other immunotherapy reports is unconfirmed.
Chen et al. 2025 [163]<br>REC02367 · Narrative discussion	HBV-positive HCC transplant recipients: pre-transplant NASH was compared for HBV reactivation and recurrence-free survival, including joint NASH–reactivation groups and reactivation strata.	Two Hangzhou transplant centers, 2015–2020. Same 274 participants as REC03247, with shared controls and different exposure definitions. Overlap with other transplant reports is unresolved.
Wang et al. 2023 [164]<br>REC00211 · Narrative discussion	Predominantly decompensated HBV cirrhosis: MAFLD was compared for mortality, first and further decompensation; metabolic subtypes were assessed within decompensated MAFLD.	No specific cross-report link identified; patient overlap is unconfirmed.
Yu et al. 2022 [167]<br>REC00101 · Narrative discussion	CHB steatosis was compared for incident diabetes by prior viral-load trajectory and HBsAg-clearance status.	Population-based Taiwanese HCC follow-up cohort; cross-report links are unconfirmed.
Oh et al. 2021 [154]<br>REC01198 · Narrative discussion	Virally suppressed CHB: CAP-defined steatosis status/grade was compared for incident HCC by stiffness-defined advanced chronic liver disease, with formal interaction testing.	Samsung Medical Center and Severance Hospital; possible overlap with other Korean antiviral cohorts, unquantified.

Report/use	Comparison and main findings	Cohort or source relationship
Xie, W.; Cao, L.; Dang, S.; Zhu, L.; Yang, D.; Zhu, Y.; Dou, X.; Gao, Z.; Chen, B.; Zhang, X.; et al. Non-alcohol fatty liver disease in a prospective national cohort of hepatitis B patients with suboptimal response to antiviral therapy. Hepatology 2023 [93], 78, S478–S479. Abstract 1371-C, The Liver Meeting, Boston, MA, USA, 10–14 November 2023. Available online: https://www.aasld.org/sites/default/files/2023-10/the_liver_meeting_boston_massachusetts_nov1_0.pdf (accessed on 25 September 2026). REC07491 · Thematic synthesis / Conference abstract	Among 90 suboptimal antiviral responders from 11 Chinese centers, CAP-defined NAFLD (39; 51 without NAFLD) accompanied higher ALT/AST and more HBV DNA >2,000 IU/mL. Conference abstract; unadjusted cross-sectional findings.	No corresponding journal report identified; publication follow-up is incomplete.

Table S1B. Ten Meta-Analyses and Their Uses

Seven contributed to outcome synthesis and are included in the 119 reports; one provided background, and two were not used because of inconsistent result reporting.

Report/use	Comparison and main findings	Source relationship
Mao et al. 2023 [37] REC00504 · Narrative discussion / Table 1	Fatty liver associations with HCC, cirrhosis, fibrosis and HBsAg clearance, stratified by treatment, diagnostic method and study design, with additional participant-data analyses.	Shares studies with Wong 2023, including Choi, Li, Mak, Wong SW and Lee.
Wong et al. 2023 [39] REC00512 · Narrative discussion / Table 1	Aggregate and individual-patient-data meta-analyses of HCC, cirrhosis, mortality and HBsAg clearance, stratified by fat-detection method and treatment.	Shares studies with Mao 2023. The aggregate analysis includes Mak, Choi, Li, Wong SW and Lee; the five IPD cohorts exclude Lee 2019.
Huang et al. 2026 [13] REC00519 · Background	Pooled mean differences and meta-regression compare HBV DNA and biochemical profiles in CHB with versus without fatty liver. Used only for background on baseline characteristics.	Overlap of constituent studies with other meta-analyses has not been fully mapped.
Li et al. 2025 [78] REC00999 · Thematic synthesis	Meta-analysis of several viral infections; only the HBV subgroup is used here. Pooled SLD associations with advanced fibrosis, cirrhosis, HCC, and antiviral response were inconclusive.	Overlap with original studies and other meta-analyses has not been fully mapped.

Report/use	Comparison and main findings	Source relationship
Zhu et al. 2025 [25]<br>REC01967 · Not used—insufficient information	Mild and moderate-to-severe steatosis versus no steatosis for treatment responses and clinical outcomes. Outcome-specific studies differed, effect directions conflicted, and groups shared controls; not used in outcome synthesis.	Shares some studies with Wong, Mao, and original cohorts.
Liu et al. 2024 [212]<br>DOI: 10.4254/wjh.v16.i3.465<br>REC02258 · Not used—insufficient information	Biochemical, viral/HBeAg responses and HCC compared across interferon and nucleos(t)ide analogue strata. Inconsistent confidence intervals, P values, and response times precluded use in outcome synthesis.	Specific overlapping studies have not been established.
Shen et al. 2024 [79]<br>REC02261 · Thematic synthesis	The pooled HCC interval spanned 1, with substantial heterogeneity; estimates varied by geography and design. Historical fatty-liver definitions differed.	Specific overlapping studies have not been established.
Jiang et al. 2021 [90]<br>REC02268 · Thematic synthesis	Pooled fibrosis and cirrhosis intervals spanned 1; 48-week viral response and ALT normalization were lower with steatosis. Fibrosis findings concerned prevalent stages.	Specific overlapping studies have not been established.
Zeng et al. 2024 [91]<br>REC02285 · Thematic synthesis	Pooled viral and biochemical responses were generally lower with NAFLD; the 24-month viral-response interval spanned 1. Study sets differed across time points.	Specific overlapping studies have not been established.
Zhu et al. 2017 [89]<br>REC07215 · Thematic synthesis	Biochemical and virological responses were lower with steatosis at 48 and 96 weeks; serological intervals spanned 1. Diagnostic methods and treatment categories were fully linked across subgroups, limiting separate attribution.	The eight constituent studies include reports by Cindoruk, Ates, Shi, Jin, Chen, Gong and Xu.

Table S1C. Reasons for Selecting the 14 Examples in Table 1

Selection required comparisons relevant to the review, adequately described groups and results, and complementary coverage of exposure definitions, metabolic burden, outcomes, disease stage, and treatment context.

Table 1 example	Reason for selection	Additional evidence for the comparison
Huang SC 2023 [35]	Compares liver fat and metabolic-condition counts against shared outcomes in one cohort.	Adalı 2024 [34] and Wang 2026 [38]: fatty-liver labels, metabolic counts and joint categories; Yun 2022 [155]: nationwide FLI-defined MAFLD, HCC and mortality (Section 8).
Lin 2024 [36]	Compares diagnostic categories and metabolic burden within a category, showing how reference groups affect interpretation.	Adalı 2024 [34]: labels and metabolic counts for HCC (Section 8); Gezer 2026 [144]: metabolic counts and biopsy fibrosis (Section 7).
Peleg 2019 [108]	Compares exposures in a shared model and illustrates interpretation of a mortality/cancer composite.	Huang SC 2025 [32]: SLD definitions and mortality; van Kleef 2021 [24]: MAFLD and a liver-event/death composite (Section 9).
Li J 2026 [109]	Compares liver fat and metabolic comorbidity in relation to tissue injury within a biopsy population.	Khalili 2021 [127]: steatohepatitis versus simple steatosis; Gezer 2026 [144]: metabolic counts within MASLD (Section 7).
Con 2024 [110]	Shows different associations with change in a shared surrogate according to the exposure definition.	Patmore 2025 [145]: serial markers; Chen 2024 [146]: fat and stiffness within biopsy-defined fibrosis stages (Section 7).

Table 1 example	Reason for selection	Additional evidence for the comparison
Huang SC 2025 [32]	Compares mortality associations as SLD definitions, populations, and reference groups change within one report.	Other Huang reports [27,33,35]: antigen, cirrhosis and HCC; Yendewa 2025 [29]: EHR-defined hepatic and mortality outcomes (Section 9).
Mak 2020 [28]	Presents antigen and fibrosis-related outcomes together, specifying fat definitions and measurement timing for each analysis.	Chang 2021 [130]: inconclusive antigen, stiffness, and HCC findings in quiescent CHB, using baseline fat and single-test HBsAg loss; Tang 2023 [140], Zhang M 2023 [141], and Yang B 2025 [114]: paired biopsies (Sections 6–7).
Chau 2025 [111]	Distinguishes virological, biochemical, and combined responses within a shared treatment source.	Li J 2020 [133]: inconclusive adjusted response estimates; its Stanford source may overlap REAL-B. Reports [131,132,139] add interferon-treated and biopsy-selected populations (Sections 6–7).
Zhang 2026 [112]	Compares hepatic and cardiovascular outcomes within one report, distinguishing coronary disease from the cardiovascular composite.	Yendewa 2025 [29]: EHR-defined hepatic and mortality outcomes; Huang R 2025 [129]: cause-specific outcomes during treatment; Yu community study: viral history and incident diabetes (Section 9).
Lin 2021 [113]	Adds renal outcomes, with the HBV subgroup reference and cross-sectional design specified.	Zhang 2026 [112]: cardiovascular outcomes; Bogdan 2025 [169]: liver–kidney background in general SLD (Section 9).
Choi 2020 [30]	Links baseline histological injury, fibrosis and fat phenotypes to subsequent clinical events.	van Kleef 2021 [24]: MAFLD analysis of the same two-center cohort; Brouwer 2015 [151]: age-adjusted steatohepatitis estimates, with possible Rotterdam-source overlap; Charatcharoenwitthaya 2017 [139]: baseline injury and treatment response (Section 7). Yoon resection cohort and immunotherapy and transplantation reports: outcomes after HCC treatment (Section 8).
Yang B 2025 [114]	Uses repeated biopsies to assess histological improvement, complementing stiffness-defined stage changes.	Tang 2023 [140] and Zhang M 2023 [141]: paired biopsies with different regimens, adjustment and regression definitions; new-onset fat findings differ. Mak 2020 [28] and Con 2024 [110]: stiffness outcomes (Section 7).
Wong YJ 2023 [39]	Provides weighted individual-patient-data estimates and comparison with aggregate-data analysis.	Mao 2023 [37]: related study-level meta-analysis; Mak 2020 [28] and Choi 2020 [30]: contributing studies (Sections 3.2, 5 and 8). A virally suppressed cohort compares HCC associations across baseline stiffness strata (Section 8).
Mao 2023 [37]	Presents study-level pooled estimates and treatment subgroups alongside participant-data analysis.	Wong YJ 2023 [39]: participant-data analysis; Adalı 2024 [34], Wang 2026 [38] and Chau 2025 [111]: different treated cohorts (Sections 5 and 8).

Table S1D. Main Findings by Outcome

A report may contribute to more than one outcome.

Clinical question	Main findings and sources	Conditions for interpretation
Histological injury and inflammatory phenotype	Some biopsy studies found less injury with fatty liver (REC00104 [45]; REC00118 [126]), others inconclusive differences (REC00031 [138]; REC00269 [46]; REC02350 [47]; REC02377 [48]), and selected MAFLD or normal-ALT populations had greater inflammation (REC00531 [49]; REC02298 [137]; REC02304 [50]). Steatohepatitis or inflammation was associated with biopsy fibrosis (REC00240 [136]; REC00183 [127]) and clinical events (REC02038 [152]; REC00077 [30]). Uncertain model values restrict REC00372 [51] to qualitative interpretation.	Cross-sectional injury and subsequent events differ; some fibrosis subgroups overlap, and denominators vary.

Clinical question	Main findings and sources	Conditions for interpretation
Metabolic labels, individual components and burden	Diabetes, hypertension, or adiposity was associated with biopsy injury (REC00087 [109]; REC00276 [142]; REC00420 [52]). Fibrosis prevalence rose with CMRF counts (REC00096 [31]), but count comparisons were inconclusive in REC00439 [144]. Injury differences between overlapping MAFLD, NAFLD and steatosis-without-metabolic-risk groups were inconclusive (REC01836 [143]). Claims-based and abdominal-obesity studies add component-versus-label comparisons (REC00117 [53]; REC02327 [54]).	Biopsy, FIB-4 and stiffness are different endpoints; diagnostic groups overlap, and adjustment models differ.
Fibrosis measurement and concurrent surrogates	Severe steatosis accompanied more stiffness-defined fibrosis (REC00284 [55]; REC02175 [56]; REC02239 [57]; REC03266 [58]). Within early biopsy stages, some studies found higher LSM with fat (REC00270 [59]; REC02357 [146]; REC02908 [60]); others were inconclusive (REC00874 [61]; REC03114 [62]; REC02569 [63]). FIB-4 findings varied with inactive CHB and HBeAg status (REC02271 [147]; REC02321 [64]); REC02237 [65] adds ALT and viral-load comparisons.	Concurrent stiffness, serum indices, tissue fibrosis and longitudinal progression differ; some thresholds and source tables remain uncertain.
Longitudinal change and treatment context	MASLD accompanied less favorable LSM-stage changes (REC00041 [145]), whereas steatosis could accompany faster LSM decline (REC00161 [110]). Treatment-response differences by BMI or fat grade were inconclusive (REC00047 [121]; REC02291 [66]). Persistent fat accompanied less biopsy improvement (REC00371 [114]). REC02727 [150] had only 8 cirrhosis events over five years; APRI trajectories differed, but event estimates remained imprecise.	Populations, treatments, fat trajectories and endpoints differ; differences in subgroup significance do not establish treatment interaction.
Disease stage and baseline event risk	MRI-measured fat was lower in HBV cirrhosis and compared with portal-hypertension surrogates (REC01055 [125]; REC02509 [67]). PAGE-B distinguished absolute HCC risk among metabolically affected patients (REC00034 [68]; REC01070 [69]).	MRI comparisons were cross-sectional. All reference participants in REC00034 [68] had metabolic problems; REC01070 [69] assessed MAFLD at last observation or HCC diagnosis.
HCC: labels, metabolic and genetic components	Within a shared source, the MAFLD estimate was near 1, while metabolic components and counts had positive associations (REC01993 [156]). Obesity had a positive association; intervals for diabetes, hypertension, and fat spanned 1 (REC00748 [157]). Univariate CMRF-count estimates were associated with HCC (REC01831 [34]). SLD had an inverse association, and multiple metabolic factors had positive associations (REC00253 [38]). Genetic analysis associated fat with lower HCC, and PNPLA3 variants and obesity with higher HCC (REC02537 [70]).	Component and count models differ; REC01993 [156] and REC02537 [70] have related male civil-service sources.
HCC: recruitment, treatment and sex	NAFLD accompanied higher HCC risk during viral suppression (REC00267 [158]). A biopsy-cohort association became inconclusive after metabolic balancing (REC00206 [71]); other obesity or fat comparisons had few events and imprecise estimates (REC01129 [72]; REC02277 [73]). Coded-SLD associations varied by sex in treated and untreated populations (REC02259 [160]).	Exposure ascertainment, metabolic adjustment, event counts and recruited sex differ; all findings are observational.
Baseline fat, fibrosis and treatment strata	Lower baseline CAP was associated with higher HCC risk; risk was highest with advanced fibrosis and no severe steatosis (REC00155 [74]). In a matched study, fatty liver accompanied lower cirrhosis/HCC risk among treated patients; corresponding untreated estimates and treated HBsAg-clearance estimates were inconclusive (REC00571 [75]).	Only baseline fat was measured. Sparse subgroup events and inconsistent model labels further limit interpretation; these findings do not establish fat loss over time.
Cirrhosis, mortality and event precision	CAP-defined fat was associated with baseline fibrosis and inversely with mortality, but available adjustment information was insufficient (REC00325 [165]). Fat–event associations were inconclusive and fibrosis associations positive (REC02306 [166]). MAFLD and diabetes were associated with cirrhosis (REC02119 [76]); MASLD estimates were inconclusive while diabetes remained associated (REC01982 [77]). Mortality associations varied by SLD definition (REC00019 [32]). REC02284 [108] reported inverse viral-load and positive death/any-cancer associations.	Baseline fibrosis, incident events, mortality and composites differ; exposure timing and adjustment matter.
Metabolic exposure duration and outcome components	Diabetes duration and repeated glycemic measurements were associated with HCC and fibrosis progression (REC01045 [120]). In a treated cohort, diabetes and metabolic-condition counts had different associations with cirrhosis, all-cause and non-liver mortality (REC01104 [129]).	Exposure scales, repeated measurements and adjustment differ; predictive superiority was not directly tested.
Meta-analyses of hepatic outcomes	HBV-subgroup pooled SLD associations with advanced fibrosis, cirrhosis and HCC were inconclusive (REC00999 [78]). The pooled HCC interval spanned 1, with variation by geography and design (REC02261 [79]).	Historical exposure definitions and constituent studies differ; primary studies overlap.

Clinical question	Main findings and sources	Conditions for interpretation
Antiviral response: fat, metabolic disease and pooled evidence	Biochemical-response associations differed between metabolic disease and steatosis/composite definitions (REC00018 [111]; REC02369 [134]). Viral suppression was lower or delayed in some reports (REC00226 [80]; REC00604 [81]; REC02208 [82]; REC02256 [83]) and similar or inconclusive in others (REC00061 [84]; REC00125 [85]; REC00744 [86]); one found similar early but lower 96-week response (REC02297 [87]). HOMA-IR was associated with later ALT normalization (REC00333 [88]); higher DNA clearance coexisted with greater baseline injury (REC00969 [135]). Meta-analyses generally found lower viral/biochemical responses with fatty liver, while serological and some timepoint findings were inconclusive (REC07215 [89]; REC02268 [90]; REC02285 [91]). REC00264 [92] compares cross-sectional ALT by viral load. Xie et al. 2023 conference report: among 90 treated CHB patients with persistent viremia, CAP-defined fat accompanied higher transaminases and more frequent HBV DNA >2000 IU/mL (REC07491 [93]; unadjusted cross-sectional comparison).	Regimens, exposures and response timing differ; REAL-B sources may overlap, and meta-analyses share constituent studies. Pooled study sets vary by timepoint; Xie applies only to persistent viremia.
Pegylated-interferon response	MASLD/steatosis accompanied lower or delayed viral responses (REC00185 [94]; REC02644 [95]); sustained-response differences in a 64-person subgroup were inconclusive (REC00622 [96]). HBsAg-clearance comparisons were also inconclusive (REC00185 [94]; REC00091 [131]; REC00463 [97]); biochemical response was assessed separately.	Monotherapy, add-on and combination regimens differ, as do baseline antigen, follow-up and denominators; some clinical and severe-fat subgroups are small.
Antigen trajectories, clearance and liver-stage outcomes	HBsAg-clearance and stiffness-progression associations varied by fat definition (REC00250 [28]; REC00544 [27]; REC02243 [98]). Dysglycemia accompanied slower HBsAg decline, and source-defined high-grade fat faster decline (REC02365 [99]). Fat differences between cured and non-cured patients were inconclusive, although baseline CAP correlated inversely with HBsAg concentration (REC02510 [100]).	Antigen concentration, change, clearance and stiffness differ. Clearance and exposure timing vary; some supplementary definitions were not fully obtained.
Hepatic, systemic and renal outcomes	Among HBsAg-positive participants, MASLD accompanied higher extrahepatic cancer and CVD risk; primary liver-cancer estimates were inconclusive (REC00122 [168]). REC00428 [112] compares hepatic and coronary outcomes. Within HBV-positive participants, NAFLD was associated with renal abnormalities (REC00207 [113]); tubular-marker comparisons were inconclusive (REC01257 [101]).	Incident events, cross-sectional renal abnormalities and urinary markers differ; HBsAg ascertainment, renal chronicity and population restrictions remain relevant.
Quiescent CHB across outcome domains	REC01092 [130] (Chang 2021): baseline CAP-defined fat had univariate HR 1.440 (95% CI 0.908–2.285) for HBsAg loss and ORs 1.085 (0.637–1.850) for stiffness-stage progression and 1.035 (0.568–1.885) for HCC; reference: the source nonsteatosis group (source Tables 2–4).	Single-test HBsAg loss, baseline CAP and univariate coefficients distinguish this comparison from Mak. Methods and table notation differ at the CAP boundary (≥248 versus >248 dB/m).
Treatment response with NAFLD	REC00259 [133] (Li 2020): NAFLD versus no NAFLD aHRs were 1.13 (95% CI 0.86–1.49) for complete viral suppression, 1.02 (0.67–1.55) for biochemical response and 1.29 (0.79–2.12) for their combined response (source Tables 3–5, model 1).	All models adjust for sex, NAFLD, metabolic syndrome, baseline HBV DNA, and FIB-4; viral/combined-response models also include regimen, and the biochemical-response model ALT. The timing of pretreatment fatty-liver diagnosis was unavailable; the Stanford and REAL-B sources may overlap.
Paired-biopsy fat trajectories	REC00305 [140] (Tang 2023): persistent and new-onset fat versus sustained absence had fibrosis-improvement aORs of 0.247 (95% CI 0.077–0.800) and 0.185 (0.058–0.588), adjusting for baseline Ishak stage (source Table S4, model 3). REC00216 [141] (Zhang 2023) reported 0.380 (0.145–0.996) for persistent and 0.936 (0.340–2.575) for new-onset fat versus persistent absence (source Table 2, model 2).	Tang: 164 participants, 104 weeks, telbivudine±adefovir, Ishak improvement. Zhang: 239 participants, 78 weeks, entecavir-based therapy, Ishak/Beijing regression; model adds baseline Ishak, age, sex, alcohol history, HBV DNA, diabetes, BMI, and week-78 detectable DNA. Model cells total 218. Both use concurrent trajectory and endpoint biopsies.

Clinical question	Main findings and sources	Conditions for interpretation
Steatohepatitis and distinct clinical endpoints	REC00327 [139] (Charatcharoenwitthaya 2017): steatohepatitis versus its absence had aORs of 10.0 (95% CI 2.08–48.5) for METAVIR ≥F2 and 3.45 (1.11–10.7) for ≥F3 (source Table 5). In the treated subset, combined week-48 viral/biochemical response was 6/14 versus 52/98 (unadjusted P=.475; source Table 6). REC00390 [151] (Brouwer 2015): age-adjusted steatohepatitis-present/absent HRs were 1.9 (0.8–4.5) for death and 1.6 (0.5–5.4) for HCC (source Table 3).	Char: F≥2 model includes steatohepatitis, ferritin, and HBV DNA; F≥3 includes steatohepatitis, hypertension, and HBV DNA. Reference includes simple steatosis and absence of fat. Brouwer: source permits alcohol exposure; 28 deaths/13 HCC events limit models, and Rotterdam cohort overlap is possible.
MAFLD in a national insurance source	REC02096 [155] (Yun 2022): MAFLD versus non-MAFLD aHRs were 1.36 (95% CI 1.31–1.41) for HCC and 1.23 (1.18–1.29) for all-cause mortality (source Table 2). The source also reports metabolic subtypes and repeated MAFLD-status comparisons.	FLI ≥30 plus metabolic criteria; model adjusts for age, sex, cirrhosis, antiviral therapy, physical activity, smoking and alcohol. Claims-based outcomes, surrogate fat, and potential Korean NHIS overlap define applicability.
Specific populations, diagnosis and mechanisms	Other eligible comparisons addressed recurrence/survival after HCC treatment, children or coinfection, baseline phenotypes, diagnostic models, exposure determinants, vascular/muscle markers and experimental mechanisms. These were used as background (individual reports in S1A–B). REC02016 [102] compares renal/CVD comorbidities among patients with established HCC.	Used for population-specific, diagnostic, or mechanistic background; the HCC comorbidity analysis concerns prevalent disease.
Information limiting use of results	11 reports had insufficient group, reference, endpoint, denominator, or numerical information for outcome synthesis; individual issues appear in S1A–B.	Eligibility decisions and reasons for non-use are retained.

Table S1E. Ninety Reports Not Used in Outcome Synthesis and the Reasons

These reports met the comparison criteria; the final column gives the individual reasons for not using them.

Report	Comparison	Reason not used in outcome synthesis
Papatheodoridis et al. 2026 [213]<br>DOI: 10.1111/jgh.70533<br>REC00013	Advanced fibrosis with versus without MASLD, separately within HBV and HCV; patient-reported outcomes also assessed.	HBV-specific fibrosis definitions and adjusted results were not extracted; patient-reported outcomes are outside this review's scope.
Sefa Sayar et al. 2023 [214]<br>DOI: 10.1016/j.ajg.2022.05.002<br>REC00032	Ultrasound steatosis in relation to GGT, HBV DNA and APRI in untreated CHB.	Adds limited information to the included longitudinal and biopsy evidence; fibrosis progression was not followed.
Hui et al. 2018 [215]<br>DOI: 10.1111/jvh.12766<br>REC00074	HBV DNA and severe-fibrosis proportions across CAP fat grades.	Adds limited cross-sectional viral-load and stiffness information to the included longitudinal CAP and biopsy reports.
Poortahmasebi et al. 2014 [216]<br>DOI: 10.7314/apjcp.2014.15.9.3879<br>REC00105	Biopsy steatosis in relation to viral markers, transaminases, necroinflammation and fibrosis.	Parallel cross-sectional comparisons; the histological synthesis already covers inconclusive and adverse associations.
Leow et al. 2023 [217]<br>DOI: 10.1111/jvh.13796<br>REC00109	Liver enzymes, stiffness and Framingham cardiovascular risk with versus without CAP-defined fat in CHB.	Cardiovascular findings concern predicted risk; observed events were prioritized. Enzymes and stiffness are covered elsewhere.

Report	Comparison	Reason not used in outcome synthesis
Minakari et al. 2009 [218]<br>DOI: 10.1097/meg.0b013e328326792e<br>REC00111	Biopsy steatosis in relation to HBeAg, HBV DNA, liver enzymes and fibrosis.	Mainly inconclusive cross-sectional viral and histological associations; included biopsy studies cover these outcomes.
Hui et al. 2022 [219]<br>DOI: 10.1111/jgh.15846<br>REC00133	MRI measures and adverse events, with serial PDFF and corrected T1 changes, in treated advanced CHB.	The abstract does not specify a PDFF–event model; MRI also captures fibroinflammation and iron, leaving fat-specific findings unclear.
Wu et al. 2024 [220]<br>DOI: 10.3760/cma.j.cn112138-20231105-00297<br>REC00137	MAFLD and advanced fibrosis, plus diabetes and advanced fibrosis within MAFLD–CHB.	Hong and Li provide label-versus-component comparisons; this additional biopsy-fibrosis comparison adds limited information.
Lai et al. 2024 [221]<br>DOI: 10.1080/17474124.2023.2298261<br>REC00179	MAFLD, survival and hepatorenal syndrome in HBV-related acute-on-chronic liver failure.	Acute-care setting; subgroup definitions, outcome-specific follow-up, and adjusted MAFLD results were not extracted.
Liu et al. 2022 [222]<br>DOI: 10.1016/j.clinre.2022.101957<br>REC00194	Fat severity, liver stiffness and fibrosis-detection accuracy within histological stages.	Biopsy/MRI-PDFF examples cover fat-related stiffness differences; additional information mainly concerns diagnostic thresholds.
Shi et al. 2008. [Prevalence and risk factors of hepatic steatosis in patients with chronic hepatitis B]. [223]<br>PMID: 18647531<br>REC00200	Steatosis, necroinflammatory grade, fibrosis stage and viral markers in 1,915 CHB patients.	Recruitment dates, sample size, and fat-group count match REC00585: Chinese- and English-language reports from the same cross-sectional biopsy source.
Chang et al. 2021 [224]<br>DOI: 10.1016/j.hbpd.2021.06.009<br>REC00201	NASH, antiviral and paired-biopsy outcomes; BMI and weight gain in relation to subsequent NASH.	Three paired-biopsy reports cover treatment-related histological change; paired samples and weight–NASH models for the weight analysis were not extracted.
Pîrvulescu et al. 2011. [The influence and prevalence of nonalcoholic steatohepatitis in chronic hepatitis B]. [225]<br>PMID: 21688564<br>REC00214	Transaminases, histological activity and fibrosis with versus without steatosis in CHB.	Parallel cross-sectional comparisons; biopsy evidence covers inconclusive fat associations and steatohepatitis-related injury.
Mi et al. 2009. [Analysis of clinical and pathological features of chronic hepatitis B with hepatic steatosis]. [226]<br>PMID: 19958639<br>REC00247	Fat presence and grade in relation to HBV DNA, inflammation, fibrosis and biochemical injury.	Lower viremia and inconclusive tissue-injury associations are covered by other viral-marker and biopsy reports.
Worland et al. 2020 [227]<br>DOI: 10.1111/liv.14344<br>REC00260	Biopsy steatohepatitis, viral-hepatitis activity and HBV viral load.	Incomplete extraction of sample composition and histological grading limits interpretation of the cross-sectional relationship between the two forms of hepatitis.

Report	Comparison	Reason not used in outcome synthesis
Shang et al. 2023 [228]<br>DOI: 10.3760/cma.j.cn501113-20220701-00362<br>REC00263	Viral markers and histological inflammation/fibrosis across CHB, CHB–MAFLD and MAFLD groups.	Group order is unclear in the English abstract; original tables are needed to confirm comparison directions.
Peng et al. 2008 [229]<br>DOI: 10.1111/j.1440-1746.2008.05478.x<br>REC00266	Steatosis, ALT and advanced fibrosis in biopsy-assessed CHB.	Included biopsy reports cover ALT, tissue injury, and inconclusive fibrosis associations.
Cindoruk et al. 2007 [230]<br>DOI: 10.1097/01.mcg.0000225586.78330.60<br>REC00274	Steatosis and sustained viral response during interferon therapy, separately by HBeAg status.	Additional information mainly concerns early peginterferon/lamivudine and HBeAg strata; this review focuses on treatment-context comparisons.
Chen et al. 2024 [231]<br>DOI: 10.1002/jmv.29613<br>REC00293	Non-invasive fibrosis-test performance under NAFLD, MASLD, MAFLD and MetALD definitions in CHB.	Additional information mainly concerns classification and diagnostic cutoffs, beyond this review's clinical-outcome discussion.
Zheng et al. 2021 [232]<br>DOI: 10.1111/apt.16595<br>REC00297	Synthesis of 54 studies relating fat to viral markers and fibrosis.	The HBV-DNA point estimate and confidence interval are inconsistent; constituent-study overlap also remains unclear.
Wen et al. 2022 [233]<br>DOI: 10.3760/cma.j.cn501113-20200628-00347<br>REC00315	NAFLD, viral/biochemical responses, APRI and stiffness changes during entecavir therapy.	Fat-related measurement effects remain unseparated from tissue-fibrosis change, limiting quantitative use.
Liu et al. 2016 [234]<br>DOI: 10.1089/jir.2015.0129<br>REC00317	BMI and computed tomography liver-to-spleen ratio, separately, in relation to early viral response in CHB–NAFLD.	This small interleukin-21 study provides only univariate BMI and liver-to-spleen results; direct CHB–SLD exposure comparisons were prioritized.
Shen et al. 2019 [235]<br>DOI: 10.1111/apt.15298<br>REC00340	Moderate/severe fat and CAP categories versus stiffness within biopsy F0–1 and F2–4 strata.	Included histological-stage comparisons cover fat effects on stiffness; additional information mainly concerns diagnostic performance.
Li et al. 2023 [236]<br>DOI: 10.3760/cma.j.cn501113-20220905-00458<br>REC00343	Viral indices, ALT, and significant fibrosis with versus without MAFLD in children.	Directions of individual comparisons are unclear in the English abstract; Chinese tables require checking.
Liang et al. 2023 [237]<br>DOI: 10.3760/cma.j.cn501113-20220124-00043<br>REC00344	Serial ALT normalization and HBV suppression with versus without NAFLD during TDF therapy.	Changing follow-up denominators and the high-sensitivity HBV-DNA subgroup were not extracted.
Han et al. 2018 [238]<br>DOI: 10.1111/apt.14843<br>REC00358	Sarcopenia and fibrosis within CHB fat and metabolic subgroups.	Sarcopenia is the primary exposure; body composition and the fibrosis scores require separate analysis.

Report	Comparison	Reason not used in outcome synthesis
Clarke et al. 2019 [239]<br>DOI: 10.1111/jvh.13147<br>REC00364	Metabolic syndrome and fat in relation to ALT, comparing Asian and non-Asian populations.	Focuses on ethnic differences in BMI thresholds and metabolic prevalence, beyond the non-obese phenotype and clinical-outcome discussion.
Chen et al. 2017 [240]<br>DOI: 10.1016/s1499-3872(16)60144-3<br>REC00406	CAP fat presence/severity, ALT normalization and HBV clearance during entecavir therapy.	Parallel CAP cohort; its cutoffs and response models add limited information to the biochemical and viral-response discussion.
Shi et al. 2008. [Hepatic steatosis: a common reason for elevated alanine aminotransferase levels in HBsAg-positive chronic hepatitis B patients with low HBV DNA loads]. [241]<br>PMID: 19032864<br>REC00413	Fat, liver enzymes, inflammation and fibrosis in low-viremia CHB selected for elevated ALT.	Normal-ALT and broader biopsy cohorts provide related comparisons; this population is restricted to low viremia with elevated ALT.
Shi et al. 2012 [242]<br>DOI: 10.3760/cma.j.issn.1007-3418.2012.04.012<br>REC00440	Fat, viral response and ALT normalization during peginterferon therapy.	Mixed-treatment and formulation details were not extracted, limiting interpretation of response differences.
Kong et al. 2025 [243]<br>DOI: 10.1016/j.acra.2025.01.003<br>REC00482	Synthesis comparing CHB-specific MAFLD/MASLD–HCC associations with broader populations.	CHB-specific syntheses already cover HCC incidence; HBV-specific populations and results for postoperative survival are unclear.
Karacaer et al. 2016 [244]<br>DOI: 10.1080/00325481.2016.1221733<br>REC00508	Fat presence/severity, necroinflammation and fibrosis in young CHB patients.	Cross-sectional biopsy findings from a young cohort; age-based recruitment alone adds limited stage-related explanation.
Lesmana et al. 2012. Prevalence of hepatic steatosis in chronic hepatitis B patients and its association with disease severity. [245]<br>PMID: 22451183<br>REC00520	Fat, viral markers, necroinflammation and fibrosis.	Parallel comparisons; adjusted estimates remain unverified, limiting quantitative use.
Nan et al. 2019 [246]<br>DOI: 10.29271/jcpsp.2019.10.937<br>REC00537	ALT and stiffness-defined fibrosis across CAP S0–S3 grades.	Adds cross-sectional ALT and stiffness distributions, requiring interpretation separately from biopsy and longitudinal progression.
Zhou et al. 2023 [247]<br>DOI: 10.1111/jvh.13872<br>REC00545	Synthesis of 88 studies relating fat to viral, biochemical, fibrosis/cirrhosis, and HCC outcomes.	The HCC direction in the text conflicts with the odds ratio; antigen abbreviations also differ. Original figures require checking.
Zhang et al. 2015 [248]<br>DOI: 10.3760/cma.j.issn.1007-3418.2015.07.009<br>REC00548	Fat and HCC in HBV cirrhosis overall and during sustained viral suppression.	Subgroup denominators and case–control references were not extracted, limiting quantitative use.
Lee et al. 2023 [249]<br>DOI: 10.1097/meg.0000000000002504<br>REC00553	Postoperative recurrence and survival in noncirrhotic NAFLD and HBV groups with or without fat.	Groups share a NAFLD reference. An entirely HBV postoperative cohort was used for a direct within-HBV fat comparison.

Report	Comparison	Reason not used in outcome synthesis
Chu et al. 2007 [250]<br>DOI: 10.1038/sj.ijo.0803479<br>REC00563	Fat grades and BMI in HBsAg-clearance cases versus persistent carriers.	Included cohorts provide longitudinal estimates for fat grades and antigen clearance.
Enomoto et al. 2023 [251]<br>DOI: 10.21873/invivo.13139<br>REC00574	Biopsy fat, insulin resistance and PNPLA3 genotype in relation to HCC.	Mainly adds PNPLA3 genotype and unadjusted events, beyond the fat-versus-metabolic HCC comparison.
Shi et al. 2008 [252]<br>DOI: 10.1111/j.1440-1746.2008.05531.x<br>REC00585	Fat, viral markers, inflammation and fibrosis in 1,915 biopsy-assessed CHB patients.	Recruitment dates, sample size, and fat-group count match REC00200, indicating the same source; histological findings parallel selected biopsy reports.
Rui et al. 2024 [253]<br>DOI: 10.1111/jvh.13942<br>REC00605	Synthesis of ten studies of biochemical, viral, and HBeAg responses during antiviral follow-up.	Potential overlap with cited original studies remains unclear.
Wang et al. 2022 [254]<br>DOI: 10.1111/jvh.13640<br>REC00611	Stiffness across fat grades within shared biopsy stages in normal-ALT CHB.	Numerical reporting is uncertain; diagnostic estimates remain unverified.
Xie et al. 2021 [255]<br>DOI: 10.1016/j.clinre.2020.03.021<br>REC00629	Shear-wave stiffness and false-positive stage classifications across fat categories within low-fibrosis strata.	Included biopsy examples cover stage overestimation; additional information mainly concerns shear-wave thresholds.
Machado et al. 2011 [256]<br>DOI: 10.1111/j.1440-1746.2011.06801.x<br>REC00641	Synthesis of fat, viral load, enzymes and histological outcomes in HBV, with a separate HCV comparison.	Overlapping constituent studies and different viremia scales complicate joint interpretation with the original studies and syntheses used.
Chen et al. 2021 [257]<br>DOI: 10.1007/s10620-020-06761-x<br>REC00654	Fat and advanced fibrosis overall; fat and significant fibrosis in noncirrhotic CHB.	Included biopsy examples cover inconclusive fat–fibrosis associations and joint steatohepatitis–fibrosis groups.
Chu et al. 2013 [258]<br>DOI: 10.1007/s10620-012-2343-9<br>REC00676	Fat, subsequent viremia, enzyme abnormalities and cirrhosis after HBsAg clearance.	Post-clearance timing matters; fat assessment, follow-up intervals and cirrhosis ascertainment were not extracted.
Altlparmak et al. 2005 [259]<br>DOI: 10.3748/wjg.v11.i20.3056<br>REC00692	Fat presence/severity, enzymes, viremia, histological activity and fibrosis in untreated CHB.	Parallel cross-sectional comparisons; other biopsy reports cover these outcomes.
Thomopoulos et al. 2006 [260]<br>DOI: 10.1097/00042737-200603000-00002<br>REC00704	Fat, fibrosis stage, and necroinflammatory grade in biopsy-assessed CHB.	Group counts and an anomalous age entry require verification; other biopsy evidence covers the cross-sectional question.
Ben Slama Trabelsi et al. 2013. [Hepatic steatosis in chronic hepatitis B: prevalence, risk factors, and impact on fibrosis and therapeutic response]. [261]<br>PMID: 24008872<br>REC00820	Fat, viral markers, fibrosis and treatment response.	Inconclusive fibrosis and response associations are covered by other biopsy and treatment reports.

Report	Comparison	Reason not used in outcome synthesis
Ayada et al. 2024 [262]<br>DOI: 10.1007/s12072-024-10639-9<br>REC00827 (evidence synthesis)	Reanalysis of 14 studies comparing HCC effects in predominantly treated versus untreated populations.	Study-level treatment categories cannot estimate within-study treatment interaction; constituent-study overlap remains unclear.
Elloumi et al. 2008. Hepatic steatosis in chronic hepatitis B: contributing factors and effect on fibrosis. [263]<br>PMID: 19213493<br>REC00836	Biopsy fat, liver enzymes, histological activity and fibrosis.	Small cross-sectional study; the biopsy synthesis covers inconclusive activity and fibrosis findings.
Chen et al. 2018 [264]<br>DOI: 10.26355/eurrev_201809_15830<br>REC00856	Biochemical and metabolic measures across fat-severity groups.	F1–F3 denote fat grades rather than fibrosis stages; this distinction and anomalous P values require checking.
Zhu et al. 2024 [265]<br>DOI: 10.1038/s41430-023-01363-y<br>REC00866	Post-transplant MAFLD, HBV recurrence, CVD, and tumor-free/overall survival.	Exposure timing and time-dependent selection remain unresolved; verified pretransplant NASH and reactivation data were used.
Persico et al. 2009 [266]<br>DOI: 10.3748/wjg.15.462<br>REC01035	Fat, BMI, diabetes and metabolic syndrome in relation to Ishak fibrosis within HBV; HCV assessed separately.	Unadjusted associations are inconclusive; the HBV multivariable model provides no adjusted fat estimate.
Kim et al. 2022 [267]<br>DOI: 10.1111/liv.15086<br>REC01102	Imaging-defined fat and obesity without diabetes, separately, in relation to biochemical response during antiviral therapy.	Parallel fat-versus-metabolic comparison; mixed regimens do not provide treatment-specific comparisons.
Mak et al. 2019 [268]<br>DOI: 10.1111/jvh.13095<br>REC01126	Fat, metabolic syndrome and ten-year fibrosis progression in HBeAg-negative CHB.	Treatment changes were not extracted, limiting interpretation alongside other serial-stiffness studies.
Seto et al. 2016 [269]<br>DOI: 10.1111/apt.13804<br>REC01127	BMI, metabolic syndrome, diabetes and CAP in relation to stiffness-defined regression during long-term antiviral therapy.	Other longitudinal reports cover lower-BMI and inconclusive CAP findings in fat-versus-metabolic comparisons.
Petta et al. 2011 [270]<br>DOI: 10.1111/j.1478-3231.2011.02453.x<br>REC01183	Fat and insulin resistance in relation to severe fibrosis, assessed separately in CHB and chronic hepatitis C.	The biopsy synthesis covers adverse fat associations and inconclusive insulin-resistance findings.
Abu Baker et al. 2025 [271]<br>DOI: 10.1177/10815589251331892<br>REC01207	Moderate/severe fat and diabetes, separately, in relation to shear-wave-defined significant fibrosis.	Model details await extraction, and estimate consistency awaits checking.
Martyn et al. 2026 [272]<br>DOI: 10.64898/2026.06.15.26355674<br>REC01218	Stiffness and HBV load across MASLD-related groups in a London CHB cohort.	The preprint's potential link to a subsequent journal report remains unclear.
Ye et al. 2020 [273]<br>DOI: 10.1007/s12072-020-10017-1<br>REC01267	MRI-PDFF fat grades, inflammation and shear-wave fibrosis stages, with biopsy validation.	Included histology/MRI-PDFF comparisons cover the measurement question; added information mainly concerns shear-wave technical estimates.

Report	Comparison	Reason not used in outcome synthesis
Liu et al. 2017. [Difference of Liver Enzymes in Different Metabolism States of Chronic Hepatitis B]. [274]<br>PMID: 28752976<br>REC01270	Enzymes across fat, glycemic, and insulin-resistance groups.	Detailed enzyme-specific metabolic profiling lies beyond the biochemical-versus-histological discussion.
Moroşan et al. 2014. Hepatic steatosis background in chronic hepatitis B and C - significance of similarities and differences. [275]<br>PMID: 25607383<br>REC01274	Inflammation and fibrosis scores across fat types in the CHB table.	Participant counts in merged cells were incompletely extracted, limiting quantitative interpretation.
Bacaksiz et al. 2021 [276]<br>DOI: 10.1111/ijcp.14899<br>REC01300	HBsAg seroconversion with mild versus moderate/severe fat in HBeAg-negative CHB.	The abstract does not specify fat-group denominators or odds-ratio references, limiting interpretation of the gradient.
Han et al. 2023 [277]<br>DOI: 10.1080/17474124.2023.2166930<br>REC01801 (evidence synthesis)	HBV-specific pooled HCC effects stratified by biopsy and other fat-detection methods.	Constituent-study overlap and study-level selection relative to existing aggregate and participant-data syntheses remain unclear.
Lin et al. 2007 [278]<br>DOI: 10.3748/wjg.v13.i12.1805<br>REC01885	Biochemical liver injury across joint HBV/fat categories using a common population reference.	The joint categories share a population reference; direct within-HBV exposure comparisons were prioritized.
Mendler et al. 2026 [279]<br>DOI: 10.1007/s10903-026-01887-y<br>REC01955	CAP and hypertension, separately, in relation to stiffness ≥6 kPa in Asian-American CHB.	Total sample size differs from the sum across centers; denominators remain unclear, and the stiffness threshold differs from this review's focus.
Khalili et al. 2021 [280]<br>DOI: 10.1093/cid/ciaa1303<br>REC02221	Simple steatosis, steatohepatitis and longitudinal ALT in HIV–HBV coinfection.	Coinfection and antiretroviral treatment require separate interpretation of ALT trajectories.
Wang et al. 2023 [281]<br>DOI: 10.1111/hepr.13854<br>REC02225	Viral markers and fibrosis indices in lean versus non-lean CHB–NAFLD, stratified by HBeAg.	Hu provides a non-obese comparison; BMI-containing fibrosis scores require separate interpretation of body-size differences.
Baclig et al. 2018. Hepatic steatosis in chronic hepatitis B: a study of metabolic and genetic factors. [282]<br>PMID: 29755645<br>REC02356	Histological activity with versus without fat, and fat grades versus fibrosis, in CHB.	Parallel biopsy comparisons; added genetic and metabolic profiling lies beyond the histological-outcome discussion.
Xiong et al. 2023 [283]<br>DOI: 10.1016/j.hbpd.2022.04.001<br>REC02390	MAFLD, recurrence-free and overall survival after resection, including an HBV–HCC subgroup.	HBV-subgroup denominators and adjusted survival estimates were not extracted; findings from all 576 patients cannot substitute for this subgroup.
Zhang et al. 2026 [284]<br>DOI: 10.1016/j.jhep.2025.10.026<br>REC02407	Immunotherapy outcomes across HBV/MASLD etiological groups.	Clinical-analysis denominators and regimens were not extracted; tissue and single-cell sample sizes do not identify the treated population.

Report	Comparison	Reason not used in outcome synthesis
Zampino et al. 2014 [285]<br>DOI: 10.4254/wjh.v6.i9.677<br>REC02415	Fat, insulin resistance and fibrosis in HBV–HCV coinfection, with monoinfection comparators.	The main analysis concerns coinfection and requires dedicated comparison of viral and metabolic settings.
John et al. 2026 [286]<br>DOI: 10.1093/infdis/jiag281<br>REC02531	HBV markers, enzymes and cytokines with versus without fat in untreated CHB.	Mainly adds cytokine profiling; HCC implications are mechanistic interpretations rather than measured clinical outcomes.
Wang et al. 2025 [287]<br>DOI: 10.1016/j.jhep.2025.01.024<br>REC03427	HBsAg loss with 1 versus at least 2 metabolic factors within MASLD in HBeAg-negative CHB.	Follow-up origin, reference group, and adjusted subgroup models were not extracted.
Wu et al. 2018 [288]<br>DOI: 10.5812/hepatmon.84182<br>REC07110	Fat grade and NAFLD activity score, separately, in relation to fibrosis in CHB with fat.	Steatohepatitis reports compare injury with fat quantity; the added activity score requires interpretation separately from a categorical diagnosis.
Karaman et al. 2013 [289]<br>DOI: 10.3906/sag-1202-96<br>REC07298	Fat, viral response and fibrosis during oral-antiviral treatment of CHB.	Response and fibrosis associations are inconclusive; timing and regimen details in this mixed-drug cohort remain unclear.
Chen et al. 2019 [290]<br>DOI: 10.3390/app9091961<br>REC07345	Fat effects on non-invasive fibrosis tests in HBV and other etiologies, with histological validation.	CHB denominators and fibrosis-stage distributions were not extracted.
Tuna et al. 2024 [291]<br>DOI: 10.5812/hepatmon-142264<br>REC07361	Fat grades and weight-change groups in HBsAg-clearance cases and controls.	Fat measurement, recalled weight-change timing, and inconsistent control counts limit quantitative interpretation.
Chon et al. 2020 [292]<br>DOI: 10.1111/jvh.13345<br>REC07402	BMI, CAP and serial stiffness improvement during tenofovir treatment of HBV cirrhosis.	Included longitudinal examples cover fat and metabolic factors, distinguishing stiffness improvement from biopsy-confirmed regression.
Patmore, Lesley Ann et al. 2023 conference abstract. RELATIONSHIP BETWEEN PRESENCE OF METABOLIC DYSFUNCTION ASSOCIATED FATTY LIVER DISEASE AND CHANGE IN LIVER STIFFNESS IN INDIVIDUALS WITH CHRONIC HEPATITIS B [293]<br>AASLD2023 PDF page 492, printed S490<br>REC07034	1,376 CHB patients, 359 with MAFLD, median follow-up 4.7 years; 682 started antivirals. MAFLD–LSM change comparisons in the full cohort and treated subgroup: increases of 25% versus 18% overall and 27% versus 15% among treated patients.	Journal report REC00041 provides serial-stiffness findings; sample size and MAFLD/MASLD definitions differ, and participant overlap is unclear.

Report	Comparison	Reason not used in outcome synthesis
Huang, Shang-Chin et al. 2023 conference abstract. PRE- EXISTING AND NEWLY-DEVELOPED METABOLIC DYSFUNCTIONS INCREASE THE RISKS OF CIRRHOSIS AND ITS COMPLICATIONS IN CHRONIC HEPATITIS B PATIENTS [294]<br>AASLD2023 PDF page 484, printed S482<br>REC07081	11,647 treatment-naive, noncirrhotic CHB patients at NTUH (2006–2021): 6,360 with metabolic dysfunction and 5,287 without. Median follow-up 5.2 years; metabolic burden, new-onset diabetes, and fat strata assessed for cirrhosis and complications.	Center, recruitment period, and longitudinal design match REC00113; the journal report updates the sample and metabolic definition.
Law, Ngai-Moh et al. 2023 conference abstract. CONCURRENT NONALCOHOLIC FATTY LIVER DISEASE (NAFLD) IN CHRONIC HEPATITIS B (CHB) HAS NO ADDITIVE EFFECT ON FIBROSIS PROGRESSION AND HEPATOCELLULAR CARCINOMA (HCC) DEVELOPMENT [295]<br>AASLD2023 PDF page 458, printed S456<br>REC07141	486 CHB patients followed for ten years; incident NAFLD assessed for fibrosis progression and HCC, with repeat elastography when indicated. Among 78 with LSM ≥9.5 kPa: NAFLD, 27 patients/2 HCC; non-NAFLD, 51/9; p = .22. The conclusion reports no increase in fibrosis stage after treatment.	Fibrosis progression is reported qualitatively, and repeat testing was clinically indicated; the HCC comparison includes only 78 patients and 11 events.
Chau, Angela et al. 2023 conference abstract. IMPACT OF FATTY LIVER (FL) ON VIROLOGIC (VR), BIOCHEMICAL (BR), AND COMPLETE RESPONSE (CR) AMONG PATIENTS WITH CHRONIC HEPATITIS B (CHB) TREATED WITH NUCLEOS(T) IDE ANALOGS (NA): A REAL- B STUDY [296]<br>AASLD2023 PDF pages 473–474, printed S471–S472<br>REC07237	REAL-B: 1,693 CHB patients (452 with fatty liver, 1,241 without) at 12 US, Taiwan and Japan centers, treated with entecavir or tenofovir. Viral, biochemical, and combined responses were compared after propensity matching.	The expanded journal report REC00018 supplies treatment-response findings; abstract responses differ, and participant overlap or supersession by the journal analysis remains unclear.
Con, D. et al. 2020 conference abstract. Epidemiology and outcomes of a chronic hepatitis B cohort with concurrent non-alcoholic fatty liver disease [297]<br>Abstract collection DOI: 10.1111/jgh.15269<br>REC07447	694 noncirrhotic CHB patients at Eastern Health (2011–2016), including 173 with ultrasound NAFLD. Fat–LSM comparisons: men, 5.9 versus 5.0 kPa, p < .001; women, 4.7 versus 4.4, p = .11. HCC: 1/173 versus 2/521, p > .99; ALT also compared.	Shares Eastern Health with REC00161; the journal report supplies serial-fibrosis findings, while sex-specific stiffness and sparse HCC events remain abstract-level.
Choi, Hannah S. J. et al. 2018 conference abstract. Non-Alcoholic Steatohepatitis Predicts Adverse Liver-Related Outcomes and Death in Chronic Hepatitis B Patients [298]<br>Abstract collection DOI: 10.1002/hep.30256<br>REC07605	1,097 CHB patients from Toronto/Erasmus tertiary centers: 910 without NASH and 187 with probable/definite NASH. Histological components and NASH within advanced-fibrosis strata compared for a clinical composite.	Same two-center cohort as REC00077; the journal report updates participants, histological estimates, and advanced-fibrosis models for clinical events.

Report	Comparison	Reason not used in outcome synthesis
Li, Jie et al. 2023 conference abstract. DIABETES MELLITUS IS AN INDEPENDENT PREDICTOR OF SIGNIFICANT INFLAMMATION AND FIBROSIS IN CHRONIC HEPATITIS B PATIENTS CONCURRENT WITH HEPATIC STEATOSIS [299]<br>AASLD2023 PDF pages 426–427, printed S424–S425<br>REC07616	869 CHB patients with biopsy-defined fat at eight Chinese centers (April 2004–October 2020), including 71 with diabetes mellitus. Diabetes: inflammation OR 3.38 (1.46–7.86); fibrosis OR 4.49 (2.08–9.72).	REC00087 supplies diabetes–injury findings; centers, recruitment, sample size, and inflammation results differ, leaving the reports' relationship unconfirmed.
Liu, Lili et al. 2023 conference abstract. THE IMPACT OF HEPATIC STEATOSIS ON RESPONSE OF ANTIVIRAL THERAPY IN CHB PATIENTS: A META- ANALYSIS [300]<br>AASLD2023 PDF page 500, printed S498<br>REC07619	Meta-analysis of 14 studies/2,591 CHB patients, searched through 22 July 2022: ALT normalization, HBV DNA suppression and HBeAg/HBsAg clearance, stratified by nucleos(t)ide analogues versus peginterferon, with 48/96-week results.	Study lists, outcome-specific denominators and complete models were unavailable; more fully reported journal syntheses were used.
Jeon, Mi Young et al. 2018 conference abstract. INFLUENCE OF HEPATIC STEATOSIS ON THE TREATMENT OUTCOMES OF ENTECAVIR AND TENOFOVIR IN PATIENTS WITH CHRONIC HEPATITIS B [301]<br>Gut 2018; 67 (Suppl. 2), A90, IDDF2018-ABS-0027<br>REC07691	334 CHB patients starting entecavir/tenofovir in 2007–2016 with transient elastography, including 146 with fat. Median follow-up 38.6 months; 303 complete viral responses and 25 HCC events. CAP–HCC p = .380; complete virological response HR .996, p = .004; HBeAg-loss HR .989, p = .031.	Cohort size, treatment period, fat group, and event counts match REC00604; the journal report defines outcomes and updates the HBeAg-loss estimate.
Peleg, Noam et al. 2019 conference abstract. Liver steatosis is a strong predictor of mortality and cancer in chronic hepatitis B regardless of viral load [302]<br>THU-341, Journal of Hepatology 2019;70:e310<br>REC07700	524 untreated CHB patients at the Rabin tertiary clinic (2007–2017), including 241 with fat and 170 biopsied; mean follow-up 6 years. Fat was inversely associated with viral load and positively with death/any cancer; joint strata numbered 55, 228, 214 and 27.	Cohort, fat group, biopsy subset and follow-up match REC02284; the journal report revises the adjusted mortality/cancer estimate.

Supplementary Table S2. Five Examples of Supporting Literature and Their Uses

These five examples of supporting literature concern serology, drug comparisons, pathological differentiation, measurement methods, and previous review interpretations.

Supporting report	Research question	Use in this review
Xu 2025 [103]	Steatosis, HBsAg clearance and seroconversion.	Supports serological outcomes; matching and interferon exposure inform the treatment-context discussion.
Dogan 2015 [104]	Tenofovir versus entecavir efficacy in CHB with steatosis.	The comparison concerns antiviral drugs and supports the treatment discussion.
Zhu 2020 [105]	Pathological differences between diagnostic groups and viral-load strata within CHB–NAFLD.	Supports pathological differentiation; comparisons concern diagnostic groups or viral-load strata.
Park 2025 [106]	CAP accuracy for detecting liver fat across fibrosis or stiffness strata.	Informs measurement error and exposure classification.
Ni 2024 [107]	Narrative review of CHB with MASLD.	Provides previous interpretations and clinical background.

Abbreviations Used in the Supplementary Tables

CHB, chronic hepatitis B; HBV, hepatitis B virus; HBsAg, hepatitis B surface antigen; HBeAg, hepatitis B e antigen; HCC, hepatocellular carcinoma; SLD, steatotic liver disease; MASLD, metabolic dysfunction-associated steatotic liver disease; MAFLD, metabolic dysfunction-associated fatty liver disease; MASH, metabolic dysfunction-associated steatohepatitis; NAFLD, nonalcoholic fatty liver disease; NASH, nonalcoholic steatohepatitis; T2DM, type 2 diabetes mellitus; CMRF, cardiometabolic risk factor; CVD, cardiovascular disease; HIV, human immunodeficiency virus; HCV, hepatitis C virus; MetALD, metabolic dysfunction and alcohol-associated liver disease; NA, nucleos(t)ide analogue; Peg-IFN, pegylated interferon; TDF, tenofovir disoproxil fumarate; pgRNA, pregenomic RNA.

AFP, alpha-fetoprotein; ALT, alanine aminotransferase; AST, aspartate aminotransferase; GGT, gamma-glutamyl transferase; BMI, body mass index; HbA1c, glycated hemoglobin; HDL, high-density lipoprotein; CAP, controlled attenuation parameter; LSM, liver stiffness measurement; MRI, magnetic resonance imaging; PDFF, proton density fat fraction; SWE, shear-wave elastography; ARFI, acoustic radiation force impulse; LiSA, liver steatosis analysis; APRI, aspartate aminotransferase-to-platelet ratio index; FIB-4, fibrosis-4 index; FLI, fatty liver index; HOMA-IR, homeostasis model assessment of insulin resistance; NAS, NAFLD activity score; NFS, NAFLD fibrosis score; PNPLA3, patatin-like phospholipase domain-containing 3; STING, stimulator of interferon genes; SAF, steatosis, activity and fibrosis score; FLIP, Fatty Liver Inhibition of Progression algorithm; P-I-R, predominantly progressive, indeterminate and predominantly regressive classification; PAGE-B, HCC risk score based on platelet count, age and sex; aMAP, HCC risk score based on age, male sex, albumin–bilirubin and platelets.

CI, confidence interval; HR, hazard ratio; OR, odds ratio; aHR, adjusted hazard ratio; aOR, adjusted odds ratio; EHR, electronic health record; IPD, individual participant data; NHIS, National Health Insurance Service (Korea); HKU, The University of Hong Kong; NTUH, National Taiwan University Hospital; GECC, Government Employees' Central Clinics; REAL-B, Real-World Evidence from the Global Alliance for the Study of HBV.
